# Paclitaxel and Cephalomannine Synergistically Induce PANoptosis in Triple-Negative Breast Cancer Through Oxygen-Regulated Cell Death Pathways

**DOI:** 10.3390/antiox14091037

**Published:** 2025-08-22

**Authors:** Xinyu Gao, Kuilin Chen, Shuhui Jia, Jiapeng Li, Huan Zhang, Yuwei Wang, Weidong Xie

**Affiliations:** 1 State Key Laboratory of Chemical Oncogenomics, Shenzhen International Graduate School, Tsinghua University, Shenzhen 518055, China; gao-xy23@mails.tsinghua.edu.cn (X.G.); ckl23@mails.tsinghua.edu.cn (K.C.); jiash24@mails.tsinghua.edu.cn (S.J.); lijp24@mails.tsinghua.edu.cn (J.L.); zhang-h24@mails.tsinghua.edu.cn (H.Z.); wang-yw24@mails.tsinghua.edu.cn (Y.W.); 2 Shenzhen Key Laboratory of Health Science and Technology, Institute of Biopharmaceutical and Health Engineering, Shenzhen International Graduate School, Tsinghua University, Shenzhen 518055, China; 3 Open FIESTA Center, Shenzhen International Graduate School, Tsinghua University, Shenzhen 518055, China

**Keywords:** triple-negative breast cancer, paclitaxel, cephalomannine, oxidative stress, programmed cell death, PANoptosis

## Abstract

Triple-negative breast cancer (TNBC) urgently requires new therapeutic strategies due to the limited efficacy of conventional treatments. Recently, PANoptosis, an integrated form of apoptosis, necroptosis, and pyroptosis, has emerged as a promising target in cancer therapy, though effective agents remain scarce. Paclitaxel, a Taxus-derived natural product, is often combined with other drugs to enhance efficacy, yet optimal combinations are limited. This study investigates the synergistic antitumor effects of paclitaxel and cephalomannine in TNBC, focusing on oxygen-regulated cell death pathways. Network pharmacology and molecular docking revealed that the combination targets multiple cell death- and inflammation-related proteins, including BCL2L1, MAPK14, SYK, TNF, and ADAM17, suggesting multi-target synergy. In vitro, the combination significantly inhibited MDA-MB-231 cell viability, proliferation, and migration, while inducing apoptosis and necrosis. Mechanistically, co-treatment markedly increased intracellular ROS levels and γ-H2AX expression, indicating oxidative stress and DNA damage, both of which were reversible by ROS inhibition. Further analysis demonstrated that the treatment activated the p38 and p53 pathways, regulated the Bax/Bcl-2 ratio, and initiated mitochondrial apoptosis. It also promoted RIPK1/RIPK3/MLKL phosphorylation and MLKL membrane translocation, triggering necroptosis, as well as upregulated NLRP3, cleaved Caspase-1, and GSDMD, inducing pyroptosis. The use of specific inhibitors partially reversed these effects, confirming the involvement of ROS-mediated PANoptosis. Similar antitumor effects were also observed in BT-549 cells, indicating the broad applicability of this combination in TNBC. MCF-10A cells exhibited mild but acceptable cytotoxicity, reflecting manageable side effects typical of chemotherapeutic agents. In vivo experiments further validated the combination’s antitumor efficacy and safety. In summary, paclitaxel and cephalomannine synergistically induce PANoptosis in TNBC through oxygen-regulated cell death pathways, offering a novel therapeutic strategy based on oxidative stress modulation by natural compounds.

## 1. Introduction

Globally, breast cancer ranks as the most frequently diagnosed malignancy among women, and both its incidence and associated mortality have shown a persistent upward trend over recent decades. Data released by the World Health Organization in 2022 indicate that breast cancer accounted for around 2.31 million newly diagnosed cases and approximately 670,000 deaths worldwide, underscoring its significant impact on women’s health [1]. The heterogeneity of breast cancer leads to significant differences in treatment and prognosis. TNBC, a subtype defined by the lack of expression of estrogen receptor (ER), progesterone receptor (PR), and human epidermal growth factor receptor 2 (HER2), is widely recognized for its aggressive clinical behavior and high likelihood of recurrence, making it one of the most difficult forms of breast cancer to manage [2,3]. As a result of the missing expression of these receptors, TNBC is unresponsive to traditional targeted and endocrine therapies, leaving chemotherapy as the primary treatment. However, TNBC often develops strong chemoresistance, especially during recurrence and metastasis, significantly reducing the efficacy of single-agent chemotherapy and creating an urgent need for multi-targeted and oxygen-regulated combination therapies [4].

Apoptosis is the most classical form of programmed cell death, regulated by the Bax/Bcl-2 signaling pathway, which controls mitochondrial outer membrane permeability, induces cytochrome c (Cytc) release, activates the caspase cascade, and ultimately leads to degradation of the cytoskeleton and nuclear structures, promoting programmed death of breast cancer cells through multiple pathways [5,6]. In recent years, growing evidence has revealed intricate interactions and mutual regulation among distinct forms of programmed cell death, including apoptosis, pyroptosis and necroptosis. These interconnected pathways form a highly integrated cell death network that supports the rationale for developing multi-targeted cancer treatment strategies. Together, apoptosis, pyroptosis and necroptosis are considered the most prominent forms of programmed cell death and constitute the foundational framework of the PANoptosis regulatory system [7]. A growing body of research indicates that modulating PANoptosis is critically involved in both the suppression and treatment of various cancers, including TNBC [8]. However, few drugs have been developed to modulate PANoptosis.

**Figure 1:**
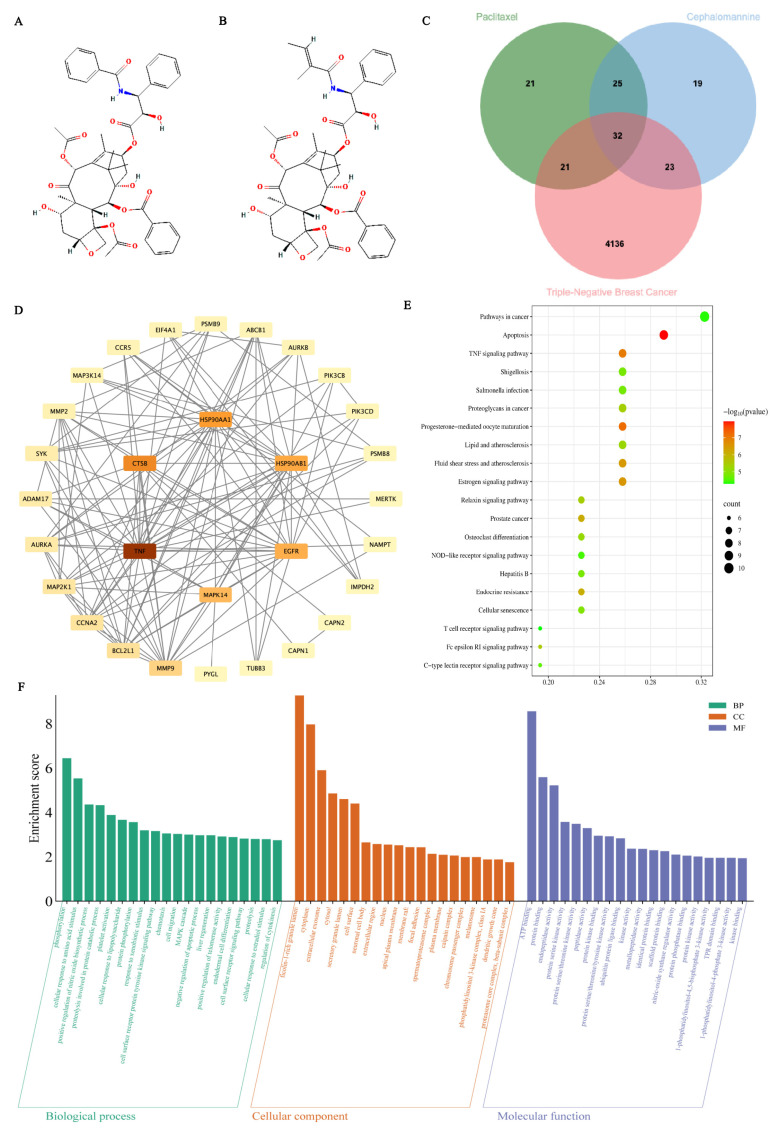
Chemical structures of paclitaxel and cephalomannine and analysis of their potential targets and enrichment results in TNBC (**A**–**F**). Chemical structure of paclitaxel (PubChem CID: 36314) (**A**). Chemical structure of cephalomannine (PubChem CID: 6436208) (**B**). Venn diagram illustrating the intersection of paclitaxel, cephalomannine, and TNBC-related genes (**C**). PPI network of the intersecting genes (**D**). KEGG pathway enrichment analysis of the intersecting genes (**E**). GO functional enrichment analysis of the intersecting genes (**F**).

Paclitaxel (Figure 1A), a taxane compound extracted from Taxus species [9,10], has become a major therapeutic drug for breast cancer and other solid tumors since its discovery in the 1960s [11]. Paclitaxel binds to tubulin to inhibit microtubule depolymerization, causing cell cycle arrest at the G2/M phase, thereby inhibiting tumor cell proliferation and inducing apoptosis. Additionally, paclitaxel has been reported to modulate redox homeostasis and trigger intracellular ROS accumulation, contributing to its antitumor effects [12]. Although paclitaxel has been marketed as an anticancer agent, its precise cellular and molecular mechanisms remain unclear, and resistance and decreased efficacy during monotherapy limit its long-term application. Cephalomannine (Figure 1B), another taxane compound derived from the leaves and branches of Taxus, has a structure similar to paclitaxel, with higher abundance and good antitumor activity. The antitumor mechanisms of cephalomannine mainly include inhibition of protein expression, induction of cell differentiation, and apoptosis [13]. However, cephalomannine alone shows limited efficacy and has not been developed as a clinical drug. Studies suggest that combination therapies can activate multiple cell death pathways to produce synergistic effects, enhancing anticancer efficacy and reducing resistance [14].

Given the critical roles of PANoptosis and oxidative stress in tumor progression and therapeutic response, and the ability of both paclitaxel and cephalomannine to modulate tumor cell proliferation and death, we hypothesized that their combination could exert enhanced antitumor effects against TNBC. This study aimed to elucidate the molecular mechanisms underlying the combination treatment, with emphasis on redox homeostasis disruption and activation of PANoptosis-related pathways. These findings are expected to provide mechanistic insight into oxygen-regulated cell death and support the development of novel therapeutic strategies for aggressive breast cancer.

## 2. Materials and Methods

### 2.1. Network Pharmacology Analysis

#### 2.1.1. SMILES Structure Acquisition, Target Prediction, and Analysis

The SMILES structures of paclitaxel and cephalomannine were obtained from the PubChem database. These SMILES structures were input into the online target prediction tool Super-PRED (https://prediction.charite.de/subpages/target_prediction.php, accessed on 2 April 2025) to predict potential targets. “Triple-Negative Breast Cancer” was used as the keyword to search TNBC-related targets in GeneCards (https://www.genecards.org, accessed on 2 April 2025), OMIM (https://www.omim.org, accessed on 2 April 2025), and DisGeNET (https://www.disgenet.com, accessed on 2 April 2025). After removing duplicates, the TNBC targets were compiled into a list for further analysis with drug targets.

#### 2.1.2. Protein–Protein Interaction (PPI) Network, Kyoto Encyclopedia of Genes and Genomes (KEGG) Pathway, and Gene Ontology (GO) Enrichment Analysis

The intersecting targets were uploaded to the STRING database (https://string-db.org, accessed on 2 April 2025), selecting “Homo sapiens” for species, to construct the PPI network. Network data processing was performed using R packages (version 2023.12.1+402) tidyverse and igraph. Visualization was carried out using Cytoscape software (version 3.10.1). Centrality parameters were calculated by the “Network Analyzer” plugin in Cytoscape to screen core targets. GO and KEGG enrichment analyses were conducted utilizing the ClusterProfiler package within the R programming environment.

#### 2.1.3. Molecular Docking

The structures of target proteins and compounds were downloaded and processed using Autodock Tools (version 1.5.7) for molecular docking. Results were visualized using PyMOL (version 2.4).

### 2.2. Cells and Reagents

Human breast cancer cells MDA-MB-231 and BT-549, as well as non-tumorigenic human mammary epithelial cells MCF-10A, were obtained from the Cell Resource Center, Shanghai Institute of Biological Sciences, Chinese Academy of Sciences(Shanghai, China).

The main reagents used included Paclitaxel (PS0036-1000, Push Biotechnology, Chengdu, China), Cephalomannine (PS2035-0025, Push Biotechnology, Chengdu, China), 3-(4,5-dimethylthiazol-2-yl)-2,5-diphenyltetrazolium bromide (MTT) Assay Kit (G020-1-1, Nanjing Jiancheng Bioengineering Institute, Nanjing, China), Mouse IL-1β Enzyme-Linked Immunosorbent Assay (ELISA) Kit (1210122, Dayu, Shenzhen, China), Mouse IL-18 ELISA Kit (MOES01225, Genie, Mt. Hope, OH, USA), Mouse TNF-α Precoated ELISA Kit (1217202, Genie, Mt. Hope, OH, USA), and Human High Mobility Group Protein B1 (HMGB-1) ELISA Kit (AE39879HU, Abebio, Wuhan, China). Cell Cycle and Apoptosis Analysis Kit (40301ES60, YEASEN, Shanghai, China), and Annexin V-FITC/propidium iodide (PI) Apoptosis Detection Kit (40302ES60, YEASEN, Shanghai, China) were also used.

Antibodies used included β-actin (A1978, Sigma-Aldrich, St. Louis, MO, USA), GAPDH (2118T, Cell Signaling Technology (CST), Danvers, MA, USA), p21 Waf1/Cip1 (E2R7A, CST, Danvers, MA, USA), NLRP3 (D4D8T, CST, Danvers, MA, USA), Gasdermin D (E9S1X, CST, Danvers, MA, USA), Cleaved Gasdermin D (Asp275) (E7H9G, CST, Danvers, MA, USA), MLKL (D2I6N, CST, Danvers, MA, USA), Phospho-MLKL (Ser358) (D6H3V, CST, Danvers, MA, USA), p38 MAPK (9212, CST, USA), Phospho-p38 MAPK (8690, CST, Danvers, MA, USA), RIP (D94C12, CST, Danvers, MA, USA), Phospho-RIP (Ser166) (D1L3S, CST, Danvers, MA, USA), RIP3 (E1Z1D, CST, Danvers, MA, USA), Phospho-RIP3 (Ser227) (D6W2T, CST, Danvers, MA, USA), Histone H2A.X (D17A3, CST, Danvers, MA, USA), Phospho-Histone H2A.X (Ser139) (20E3, CST, Danvers, MA, USA), DRP1 (F0328, Selleck, Shanghai, China), Phospho-DRP1 (Ser637) (F0594, Selleck, Shanghai, China), Caspase-8 (F0067, Selleck, Shanghai, China), PARP (F0148, Selleck, Shanghai, China), Cleaved PARP (Asp214) (F0136, Selleck, Shanghai, China), Caspase-3 (F1080, Selleck, Shanghai, China), IL-18 (F2146, Selleck, Shanghai, China), NLRP3 (F0335, Selleck, Shanghai, China), and HMGB1 (A5052, Selleck, Shanghai, China). Secondary antibodies included Goat Anti-Mouse IgG (#7076S, CST, Danvers, MA, USA) and Goat Anti-Rabbit IgG (#7074P2, CST, Danvers, MA, USA).

Other reagents included Pre-stained Protein Marker (26617, ThermoFisher Scientific, Waltham, MA, USA), WB ECL Chemiluminescence Reagent (32106, ThermoFisher Scientific, Waltham, MA, USA), SDS-PAGE Sample Buffer (ZS306, YEASEN, Shanghai, China), Quick PAGE Gel Preparation Kit (10%, pg112, YEASEN, Shanghai, China), Reactive Oxygen Species (ROS) Assay Kit (S0033S, Beyotime Biotechnology, Shanghai, China), YO-PRO-1/PI Apoptosis and Necrosis Detection Kit (C1075M, Beyotime Biotechnology, Shanghai, China), JC-1 (C2005, Beyotime Biotechnology, Shanghai, China), Luminescent ATP Detection Assay Kit (K2040, APExBIO, Houston, TX, USA), Hematoxylin-Eosin (HE) Staining Kit (G1003, Servicebio, Wuhan, China), 20× Citrate Antigen Retrieval Solution (G1202, Servicebio, Wuhan, China), Bovine Serum Albumin (BSA, GC305010, Servicebio, Wuhan, China), and DAB Chromogenic Kit (G1212, Servicebio, Wuhan, China). ALT Determination Kit (232821, BIOSINO, Beijing, China), AST Determination Kit (231431, BIOSINO, Beijing, China), UREA Determination Kit (241551, BIOSINO, Beijing, China), CRE Determination Kit (240932, BIOSINO, Beijing, China), TG Determination Kit (248131, BIOSINO, Beijing, China), CHO Determination Kit (232091, BIOSINO, Beijing, China).

### 2.3. Cell Culture

MDA-MB-231, MCF-10A cells were cultured in Dulbecco’s Modified Eagle Medium (DMEM) supplemented with 10% FBS and 1% Penicillin-Streptomycin in a humidified incubator at 37 °C with 5% CO_2_. BT-549 cells were cultured in 1640 supplemented with 10% FBS and 1% Penicillin-Streptomycin in a humidified incubator at 37 °C with 5% CO_2_

### 2.4. MTT Assay

Log-phase cells were collected using 0.25% trypsin, centrifuged at 800 r/min for 3 min at 4 °C, resuspended in the appropriate culture medium for each cell line, and seeded in 96-well plates at 5 × 10^3^ cells per well. After 12 h, cells were treated with paclitaxel or cephalomannine at final concentrations of 1 ng/mL, 5 ng/mL, or 10 ng/mL, dissolved in Dimethyl Sulfoxide (DMSO). An equal volume of DMSO was administered to the control group as a vehicle treatment. After 48 h, MTT reagent was added to each well and the cells were incubated for an additional 2 to 3 h. The resulting formazan crystals were subsequently solubilized using DMSO. Absorbance was then recorded at 490 nm using a microplate reader.

### 2.5. Clonogenic Assay

Cells were seeded into 6-well plates at a density of 500 cells per well and allowed to adhere overnight. On the following day, cells were treated with paclitaxel, cephalomannine (both at 1 ng/mL). The treatment was maintained for 15 days, during which the medium containing the drugs was refreshed every 3 days. At the end of the incubation period, cells were fixed with 4% paraformaldehyde for 15 min and stained with 0.1% crystal violet for 30 min at room temperature. Colonies were counted under a microscope, and images were captured for quantification.

### 2.6. Cell Migration and Invasion Assays

For the migration assay, the upper chambers were left uncoated. For the invasion assay, the upper surface of the inserts was coated with 50 μL of Matrigel (1:8 dilution in serum-free medium) and incubated at 37 °C for 1 h to allow gel solidification. Cells were pre-treated with paclitaxel, cephalomannine, or their combination (each at 1 ng/mL) for 24 h, then harvested and resuspended in serum-free DMEM. A total of 5 × 10^4^ cells in 200 μL serum-free medium were seeded into the upper chamber of each insert. The lower chambers were filled with 600 μL DMEM containing 10% FBS as a chemoattractant. After incubation at 37 °C for 24 h (migration) or 48 h (invasion), non-migrated/non-invaded cells on the upper side of the membrane were removed with a cotton swab. The membranes were washed with PBS, and the migrated/invaded cells on the lower side were fixed with 4% paraformaldehyde for 15 min and stained with 0.1% crystal violet for 30 min at room temperature. Images were captured using an inverted microscope, and cells were counted in five randomly selected fields per well. Quantitative analysis was performed using ImageJ software (Version 2.3.0).

### 2.7. Western Blot Analysis

Cells were seeded in 6-well plates at a density of 1 × 10^5^ cells per well and allowed to adhere overnight. Cells were then exposed to paclitaxel and cephalomannine at a final concentration of 1 ng/mL for 48 h prior to protein isolation. After treatment, the cells were harvested, rinsed with cold PBS, and lysed on ice for 15 min using RIPA buffer supplemented with protease and phosphatase inhibitors. The lysates were centrifuged at 12,000 rpm for 10 min at 4 °C to obtain the supernatants, which were used for subsequent protein concentration determination using a Bicinchoninic Acid Assay (BCA) assay. Equal amounts of protein were mixed with loading buffer and boiled at 100 °C for 15 min to denature the proteins. Proteins were separated by SDS-PAGE and transferred onto Polyvinylidene Fluoride (PVDF) membranes. Membranes were blocked with 5% non-fat milk or BSA in TBST for 1 h at room temperature and incubated overnight at 4 °C with specific primary antibodies. After washing, membranes were incubated with HRP-conjugated secondary antibodies for 1 h at room temperature. Protein bands were visualized using enhanced chemiluminescence (ECL) and imaged with a gel documentation system.

### 2.8. ELISA

After treatment with paclitaxel and cephalomannine (both at 1 ng/mL) for 48 h, the cell culture supernatants were collected. According to the manufacturers’ instructions, ELISA assays were performed to detect the expression levels of cytokines IL-1β, IL-18, TNF-α, and the DAMP molecule HMGB1. Absorbance was measured using a microplate reader, and protein concentrations were calculated based on standard curves.

### 2.9. Cell Cycle and Apoptosis Analysis

Cells were seeded in 6-well plates at a density of 1 × 10^5^ cells per well and allowed to adhere overnight. The cells were then treated with paclitaxel and cephalomannine (1 ng/mL each) for 48 h. For cell cycle analysis, cells were harvested, washed with cold PBS, and fixed in 70% ethanol at 4 °C overnight. After fixation, cells were washed and stained with PI containing RNase A for 30 min at room temperature in the dark. Cell cycle distribution was analyzed by flow cytometry (Beckman Coulter Life Sciences, USA) and quantified using FlowJo software (verison 10.8.1). For apoptosis detection, after harvesting and washing, cells were resuspended in binding buffer and incubated with Annexin V-FITC (5 μL) and PI (5 μL) for 15 min in the dark at room temperature. Stained cells were immediately analyzed by flow cytometry. To distinguish apoptotic and necrotic cell populations, YO-PRO-1/PI staining was performed. After drug treatment, cells were incubated with 1 μM YO-PRO-1 and 1 μg/mL PI for 20 min at room temperature in the dark, and then observed under a fluorescence microscope. Images were captured and fluorescence intensity was quantified using ImageJ software.

### 2.10. ROS Detection

Cells were seeded in 6-well plates at a density of 1 × 10^5^ cells per well and incubated overnight. Cells were then treated with paclitaxel and cephalomannine (1 ng/mL each) for 48 h. After treatment, cells were washed twice with PBS and incubated with 10 μM DCFH-DA (diluted 1:1000 in serum-free DMEM) at 37 °C for 30 min in the dark. Following incubation, cells were washed three times with PBS to remove excess probe, and intracellular ROS levels were immediately observed under a fluorescence microscope using a FITC filter set. Images were acquired using consistent exposure settings across all samples. Quantification of fluorescence intensity was performed using ImageJ software by calculating the integrated density normalized to the cell area (IntDen/Area), with at least five fields analyzed per group. For the ROS inhibitor experiment, cells were pretreated with 5 mM N-acetylcysteine (NAC) for 2 h before drug administration. After NAC pretreatment, the same procedures as described above were followed for drug treatment and ROS detection.

### 2.11. Mitochondrial Membrane Potential

The mitochondrial membrane potential (ΔΨm) was evaluated using the JC-1 fluorescent probe. MDA-MB-231 cells were seeded into 6-well plates and cultured until adherence. Cells were subsequently treated with paclitaxel and cephalomannine, each at a concentration of 1 ng/mL, for 48 h. Following drug exposure, the culture medium was removed and replaced with JC-1 working solution (diluted 1:200 in complete medium). After incubation at 37 °C for 30 min, cells were collected by enzymatic digestion and centrifugation, and then subjected to flow cytometric analysis.

### 2.12. ATP Assays

Cells were seeded in 6-well plates at a density of 1 × 10^5^ cells per well and cultured overnight. Cells were then treated with paclitaxel and cephalomannine (1 ng/mL each) for 48 h. For intracellular ATP detection, culture medium was removed and 200 μL of pre-chilled lysis buffer was added. After complete lysis on ice, samples were centrifuged at 12,000× *g* for 10 min at 4 °C, and the supernatant was collected for analysis. For extracellular ATP, culture supernatants were centrifuged under the same conditions and used directly. In both cases, 100 μL of detection reagent was added and incubated at room temperature for 3–5 min. Then, 20 μL of sample or standard was added, mixed, and briefly equilibrated before luminescence was measured using a chemiluminescence plate reader.

### 2.13. Animal Experiments

Female BALB/c-nude mice (3–4 weeks old, ~18 g) were randomly divided into four groups (*n* = 7 per group). MDA-MB-231 cells in the logarithmic phase were digested, centrifuged, resuspended in Matrigel, adjusted to 2 × 10^7^ cells/mL, and injected subcutaneously (2 × 10^6^ cells/mouse) to establish xenograft models. Treatment groups received paclitaxel (2.9 mg/kg), cephalomannine (2.9 mg/kg), or combination, by gavage with soybean oil as the solvent. Control group mice received soybean oil alone. Dosing volume was 0.1 mL/10 g body weight.

At the end of the study, mice were fasted (but given water) for 6 h, anesthetized with urethane, and blood samples were collected. Major organs and tumors were harvested for weighing, fixation, or storage at −80 °C.

### 2.14. HE Staining

Tumor samples were paraffin-embedded, sectioned, and subjected to standard hematoxylin-eosin staining following dewaxing and gradient ethanol dehydration. After mounting with neutral resin, tissue sections were examined and photographed using a light microscope.

### 2.15. Immunohistochemistry

Paraffin-embedded tumor sections were dewaxed and subjected to antigen retrieval using EDTA buffer (pH 8.0), followed by blocking with 3% BSA. After overnight incubation with primary antibodies at 4 °C, sections were treated with HRP-labeled secondary antibodies. DAB served as the chromogenic substrate, and hematoxylin was applied for nuclear staining. Finally, the slides were dehydrated, sealed, and observed under a microscope.

Immunohistochemical staining was evaluated using the H-SCORE method. Five random areas from each section were analyzed with Image-Pro Plus 6.0 software. The score was calculated as: 1 × (percentage of weak staining) + 2 × (moderate) + 3 × (strong), with a total range of 0 to 300, reflecting both intensity and positive cell proportion.

### 2.16. Transmission Electron Microscopy (TEM) and Scanning Electron Microscopy (SEM)

Cells were treated with 1 ng/mL paclitaxel and cephalomannine for 48 h. For TEM, cells were fixed with 2.5% glutaraldehyde, post-fixed with osmium tetroxide, dehydrated, embedded, sectioned (60–80 nm), stained, and imaged. For SEM, fixed cells were dehydrated, processed with isoamyl acetate, dried, gold-coated, and examined under SEM.

### 2.17. Statistical Analysis

All data are presented as mean ± standard deviation (SD). Statistical analysis was performed using GraphPad Prism (verison 10). One-way ANOVA followed by Newman-Keuls post hoc test was used for group comparisons. A *p*-value < 0.05 was considered statistically significant.

## 3. Results

### 3.1. Network Pharmacology Analysis of Paclitaxel and Cephalomannine

A Venn analysis of genes related to paclitaxel, cephalomannine, and TNBC identified 32 candidate genes (Figure 1C). These genes are linked to various pathways regulating cell survival, proliferation, and programmed cell death. A PPI network (Figure 1D) was constructed to clarify the functional relationships of these candidate targets. Analysis showed that TNF, MAPK14, SYK, BCL2L1, and ADAM17 were core nodes with high connectivity and were closely associated with apoptosis, pyroptosis, and necroptosis. Among them, TNF, as a key inducer of necroptosis, had the highest degree of connectivity and may regulate inflammation and cell death signaling. ADAM17, an upstream regulator of the TNF signaling pathway, may enhance TNF signal amplification. MAPK14 and SYK play important roles in inflammation activation and signal transduction and may inhibit tumor cell proliferation by mediating pyroptosis. BCL2L1 is a critical anti-apoptotic protein that helps regulate the equilibrium between cell survival and apoptosis. These results suggest that paclitaxel and cephalomannine may regulate the programmed death of TNBC cells through multi-target synergy.

KEGG enrichment analysis identified major signaling pathways associated with the candidate targets (Figure 1E). The most significantly enriched pathways included the apoptosis pathway and the TNF signaling pathway, indicating that paclitaxel and cephalomannine may directly regulate apoptosis and inflammatory responses to enhance programmed death of TNBC cells. The candidate targets also participated in cancer-related pathways, MAPK signaling, and NOD-like receptor signaling pathways, which are involved in cellular stress, immune regulation, and inflammatory cell death, further supporting that paclitaxel and cephalomannine may effectively inhibit TNBC cell proliferation and migration via a synergistic multi-target, multi-pathway mechanism.

GO enrichment analysis highlighted the biological functions associated with the candidate targets (Figure 1F). In the Biological Process (BP) category, the targets were mainly enriched in pathways related to apoptosis, inflammation, cell cycle control, and signal transduction, suggesting that paclitaxel and cephalomannine may induce programmed cell death and suppress uncontrolled proliferation in TNBC through multiple mechanisms. For the Cellular Component (CC) category, the majority of targets were associated with cytoplasmic, nucleus, and membrane-associated complexes, suggesting that their functions rely on membrane receptor signaling and intracellular regulatory pathways. In the Molecular Function (MF) category, the targets were primarily enriched in protein binding, enzyme activity regulation, and DNA binding, implying that the two drugs may exert multi-target regulation by modulating key enzymes and transcription factors.

### 3.2. Molecular Docking of Paclitaxel and Cephalomannine with Core Targets 

**Figure 2:**
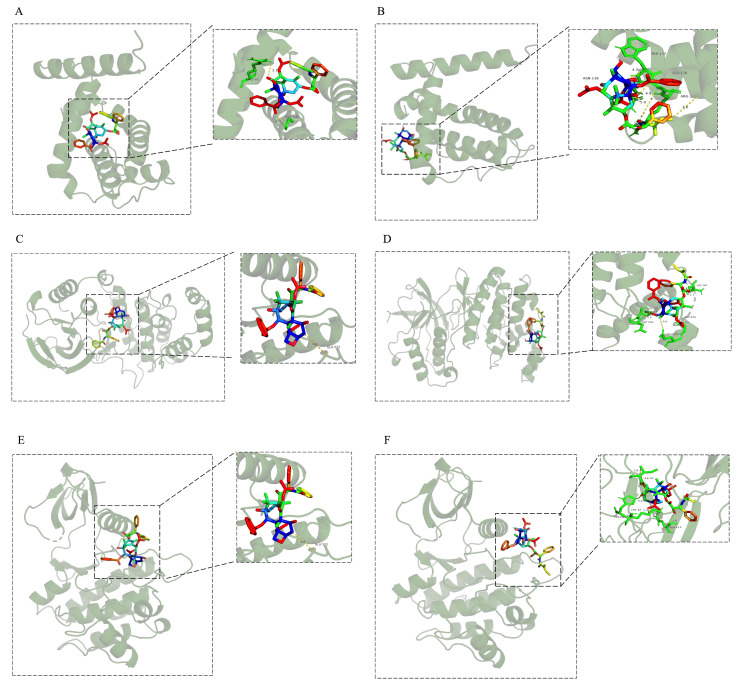

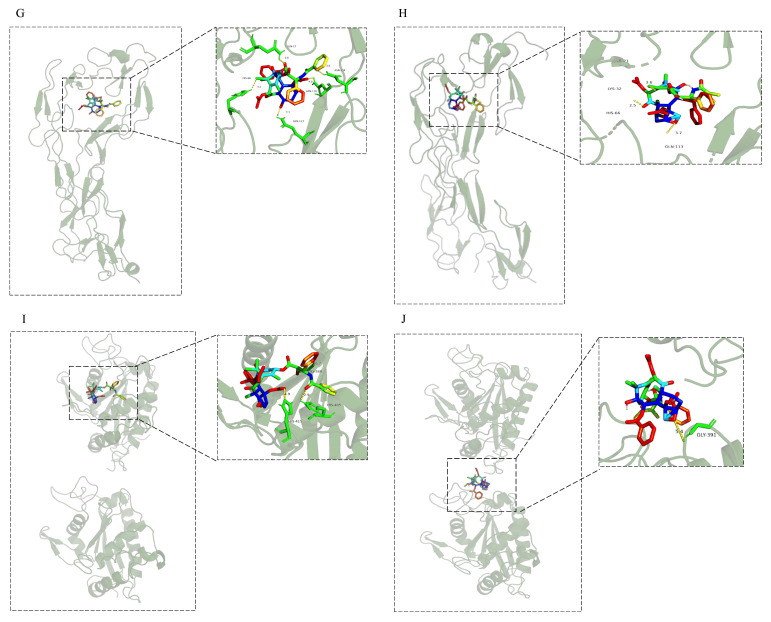
Molecular docking results of paclitaxel and cephalomannine with the active sites of BCL2L1, MAPK14, SYK, TNF, and ADAM17 proteins (**A**–**J**). Docking of paclitaxel with BCL2L1 (**A**). Docking of cephalomannine with BCL2L1 (**B**). Docking of paclitaxel with MAPK14 (**C**). Docking of cephalomannine with MAPK14 (**D**). Docking of paclitaxel with SYK (**E**). Docking of cephalomannine with SYK (**F**). Docking of paclitaxel with TNF (**G**). Docking of cephalomannine with TNF (**H**). Docking of paclitaxel with ADAM17 (**I**). Docking of cephalomannine with ADAM17 (**J**).

Network pharmacology analysis identified several core targets that may participate in the regulation of programmed cell death. To further verify the mechanisms of action of paclitaxel and cephalomannine at the molecular level, molecular docking studies were performed. Molecular docking is a widely used computational simulation method that predicts the binding modes and binding affinities between small molecule drugs and target proteins, thus providing theoretical support for elucidating the drug action mechanisms [15]. Five important targets with high connectivity (BCL2L1, MAPK14, SYK, TNF, and ADAM17) were selected for molecular docking analysis (Figure 2). The results showed that the binding energies of paclitaxel to these targets were BCL2L1 (−7.4 kcal/mol), MAPK14 (−7.9 kcal/mol), SYK (−8.6 kcal/mol), TNF (−9.3 kcal/mol), and ADAM17 (−9.5 kcal/mol), indicating strong binding affinity. Cephalomannine exhibited better binding energies than paclitaxel at BCL2L1 (−7.9 kcal/mol), MAPK14 (−9.0 kcal/mol), and SYK (−8.7 kcal/mol), while slightly lower binding energies were observed at TNF (−7.4 kcal/mol) and ADAM17 (−8.2 kcal/mol) (Table 1).

These key targets play crucial roles in various programmed cell death pathways: BCL2L1 is involved in mitochondrial-mediated apoptosis; MAPK14 and SYK regulate inflammatory responses and are associated with pyroptosis; TNF and ADAM17 are key molecules in necroptosis signaling. The molecular docking results also suggested that paclitaxel and cephalomannine may exert complementary binding capacities at different targets, achieving synergistic regulation across apoptosis, pyroptosis, and necroptosis pathways to enhance antitumor effects.

Based on the network pharmacology analysis, paclitaxel and cephalomannine may regulate multiple signaling pathways involved in PANoptosis, including apoptosis, pyroptosis, and necroptosis. To further validate the potential synergistic effects and their regulatory roles in programmed cell death, we conducted comprehensive in vitro and in vivo experiments. These included cell viability assays, apoptosis analysis, pyroptosis marker detection, and examination of necroptosis-related signaling, aimed at fully elucidating the unique synergistic antitumor mechanisms of paclitaxel and cephalomannine.

### 3.3. Effects of Paclitaxel and Cephalomannine Combination on MDA-MB-231 Cell Proliferation, Apoptosis, and Migration/Invasion

**Figure 3:**
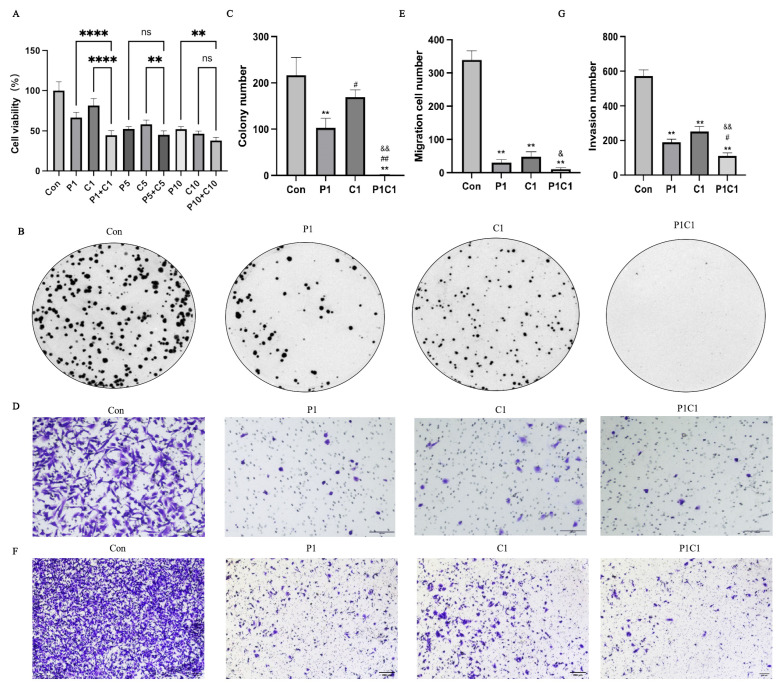
Effects of paclitaxel and cephalomannine combination treatment on MDA-MB-231 cell viability, colony formation, migration, and invasion (**A**–**G**). Effects of single and combined treatments of paclitaxel and cephalomannine on MDA-MB-231 cell viability (**A**). Representative photographs and statistical evaluation of colony formation (**B**,**C**). Images and quantification of cell migration in the Transwell assay (**D**,**E**). Representative photographs and statistical evaluation of colony formation (**F**,**G**). P represents paclitaxel (P1 = 1 ng/mL); C represents cephalomannine (C1 = 1 ng/mL). Data are expressed as mean ± standard deviation (SD) from biological replicates. For cell viability, migration, and invasion assays, *n* = 5; for colony formation assay, *n* = 3, ** *p* < 0.01 vs. Con, ## *p* < 0.01 vs. P1, # *p* < 0.05 vs. P1, && *p* < 0.01 vs. C1, & *p* < 0.05 vs. C1, **** *p* < 0.0001, ns indicates not significant.

We began with in vitro validation. MTT assays were used to assess the effects of various drug concentration combinations on MDA-MB-231 cell viability (Figure 3A). After 48 h, both paclitaxel (P1) and cephalomannine (C1) alone significantly inhibited cell viability, and their combination (P1C1) produced an even greater reduction, indicating a notable synergistic effect. However, increasing the dose (P5C5 and P10C10) did not further enhance the inhibitory effect compared to P1C1, suggesting that low-dose treatment was sufficient to achieve maximal suppression.

To balance experimental efficacy and drug toxicity, P1C1 was selected as the optimal dose for subsequent analyses and validation. The synergistic interaction between paclitaxel and cephalomannine was quantitatively evaluated using the Combination Index (CI), a widely accepted metric based on the Chou–Talalay method, which applies the median-effect principle to determine drug interactions. In this model, CI < 1 indicates synergism, CI = 1 indicates an additive effect, and CI > 1 indicates antagonism. CI values were calculated using CompuSyn software (version 1.0.1) (Table 2). Results showed that when the total concentration of paclitaxel and cephalomannine ranged from 2 to 20 ng/mL, all CI values were less than 1 (range: 0.09–0.72), clearly indicating synergy. Notably, the synergistic effect was more pronounced at lower concentrations (CI < 0.2).

Colony formation assays further supported the inhibitory effect of the combined treatment on MDA-MB-231 cell growth (Figure 3B,C). Both P1 and C1 alone significantly decreased colony numbers compared to the control, while P1C1 nearly abolished colony formation, indicating a substantial reduction in proliferative and survival capacity.

To assess impacts on tumor cell malignancy, we conducted Transwell assays. Both paclitaxel and cephalomannine alone suppressed MDA-MB-231 cell migration and invasion, with the P1C1 exhibiting the strongest inhibitory effect (Figure 3D–G). Quantitative analysis showed that the P1C1 treatment reduced migrated cells by ~90% and invaded cells by ~80% compared to controls, suggesting a significant impairment of metastatic and invasive behavior in addition to growth inhibition.

**Figure 4:**
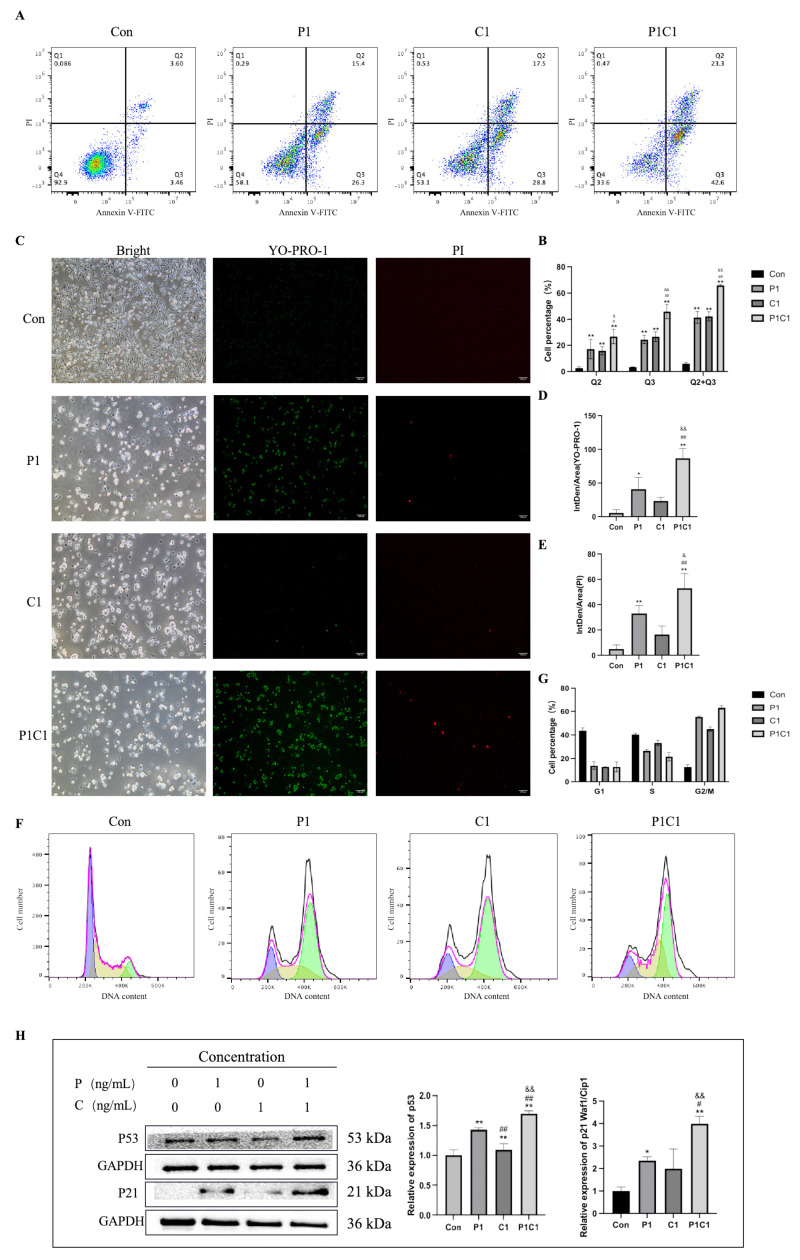
Effects of paclitaxel, cephalomannine, and their combination on cell cycle and apoptosis in MDA-MB-231 cells (**A**–**H**). Flow cytometry analysis of apoptosis under different treatment conditions (**A**,**B**). YO-PRO-1/PI dual staining assay detecting apoptosis under different treatments (**C**–**E**). Flow cytometry analysis of cell cycle distribution under different treatments (**F**,**G**). Western blot analysis and quantification of p53 and p21 expression levels (**H**). P represents paclitaxel (P1 = 1 ng/mL); C represents cephalomannine (C1 = 1 ng/mL). Data are expressed as mean ± standard deviation (SD) from biological replicates (*n* = 3). ** *p* < 0.01 vs. Con, * *p* < 0.05 vs. Con, ## *p* < 0.01 vs. P1, # *p* < 0.05 vs. P1, && *p* < 0.01 vs. C1, & *p* < 0.05 vs. C1.

After confirming that the combination of paclitaxel and cephalomannine significantly inhibited MDA-MB-231 cell viability and induced cell death, we further investigated whether this effect was mediated by typical programmed cell death mechanisms. First, Annexin V-FITC/PI dual staining flow cytometry was used to analyze apoptosis rates. The results showed that both P1 and C1 significantly induced apoptosis compared with the control group. In the combination group, apoptosis was further increased early and late apoptosis, which was significantly higher than that of the single-treatment groups (Figure 4A,B).

To further confirm whether this cell death was related to programmed cell death, YO-PRO-1/PI dual staining was used to detect the type and extent of cell death (Figure 4C–E). The results showed that single drug treatments induced a certain degree of early apoptosis (green fluorescence) and late apoptosis/necrosis (red fluorescence), while the fluorescence intensity was significantly enhanced in the combination group, indicating a substantial increase in cell death. In the P1C1 group, both green and red fluorescence were simultaneously enhanced, suggesting that aggravated apoptosis may have been accompanied by secondary necrosis. These findings align with the results from the MTT and colony formation assays and further confirmed the synergistic effect of the two drugs in inducing programmed cell death.

Considering that apoptosis is often closely associated with cell cycle arrest, which triggers cell cycle checkpoints to halt progression, we further explored the impact on the cell cycle. When repair is not possible, cells initiate apoptosis to prevent the transmission of genetic defects [16]. We analyzed cell cycle distribution using PI staining and flow cytometry (Figure 4F,G). The results showed that P1 treatment significantly arrested cells at the G2/M phase, while C1 mainly caused accumulation in the S phase, indicating that the two drugs may interfere with cell cycle regulation through different mechanisms. Under the P1C1 combination treatment, G2/M phase arrest was further enhanced, and the proportion of cells in the S phase was significantly reduced. This suggests that the combination treatment exerted an additive effect in blocking cell cycle progression, preventing cells from entering mitosis, and thereby synergistically promoting cell inhibition and death. We further examined the expression levels of the key regulatory proteins p53 and p21 using Western blot. The results showed that both P1 and C1 significantly upregulated p53 and p21 expression, and their expression levels were further elevated in the combination group (Figure 4H).

### 3.4. Ultrastructural Evidence Supporting That Paclitaxel and Cephalomannine Combination Induces Multiple Types of Programmed Cell Death

Based on the previous results showing that paclitaxel and cephalomannine combination in suppressing proliferation and promoting cell death in MDA-MB-231 cells (Figure 3 and Figure 4), we further investigated whether non-classical forms of programmed cell death were also involved. Notably, large numbers of PI-positive signals were observed in the YO-PRO-1/PI staining assay after combination treatment, and similar results were obtained from flow cytometry, suggesting that in addition to apoptosis, secondary necrosis or other non-classical programmed cell death forms might be present. Since flow cytometry and staining assays alone cannot precisely distinguish different cell death types, we further performed ultrastructural analysis using TEM.

**Figure 5:**
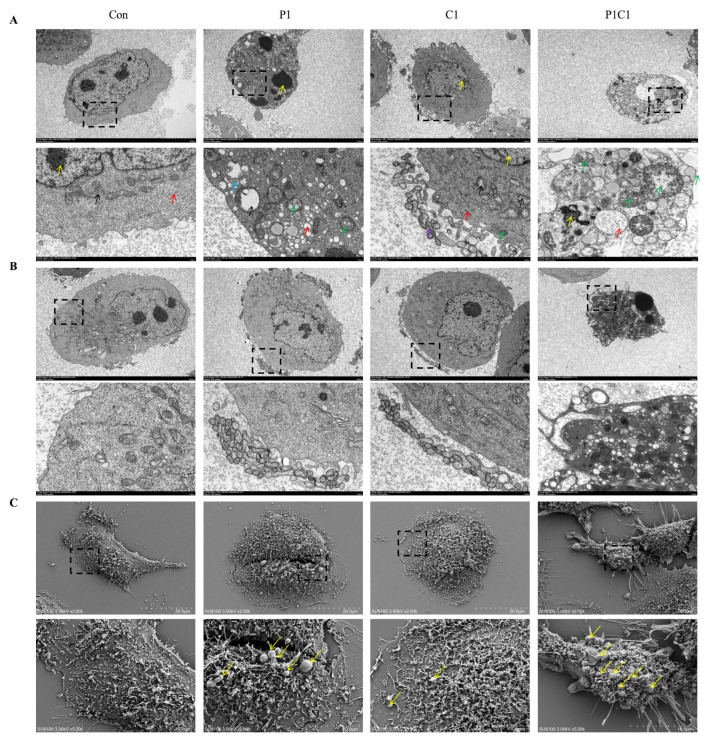
Ultrastructural and surface morphological changes in MDA-MB-231 cells following combined paclitaxel and cephalomannine treatment (**A**–**C**). TEM observation of plasma membrane morphological changes (**A**,**B**). SEM images showing changes in cell surface morphology (**C**). Dotted squares indicate the regions that were enlarged in the corresponding images.

TEM observations (Figure 5A) showed that control group cells exhibited intact structure, clear nucleoli, continuous nuclear membranes, regular mitochondrial morphology with intact cristae, and no endoplasmic reticulum (ER) swelling (yellow, black, and red arrows). P1 group cells displayed significant damage, including chromatin condensation, mitochondrial membrane rupture, cristae fragmentation, ER swelling, increased autophagosomes (green arrows), and some lysosomes (blue arrows), consistent with typical apoptotic morphology. In the C1 group, cells showed milder damage, mitochondrial cristae disruption with intact nucleoli and ER, and abundant secretory vesicles. In contrast, the P1C1 combination group showed the most severe damage, including highly condensed chromatin, loose cytoplasm, abundant autophagolysosome formation, and severe ER swelling with vacuolization, indicating pronounced programmed cell death and necrosis.

Moreover, in addition to chromatin condensation and autophagolysosome accumulation, severe ER swelling and cell membrane rupture were observed under TEM in the combination group, which are atypical for apoptosis, suggesting that other forms of programmed cell death may be activated. Combined with network pharmacology results, these findings strongly support that the two drugs may jointly act on multiple key targets of programmed cell death pathways. In addition to apoptosis regulators like BCL2L1, targets such as MAPK14, SYK, and TNF, involved in inflammation and pyroptosis/necroptosis pathways, were significantly enriched (Figure 1). Notable enrichment in the TNF and NOD-like receptor signaling pathways suggested that the combination might activate non-classical cell death mechanisms such as pyroptosis and necroptosis through multi-target synergy, thereby exacerbating tumor cell damage and enhancing anticancer efficacy.

To further verify this hypothesis, we observed additional fields of the same sample set under TEM (Figure 5B), focusing on plasma membrane morphological changes such as blebbing, shrinkage, and bubble-like protrusions, which are hallmarks of pyroptosis. The control group showed smooth, intact membranes. In contrast, both P1 and C1 groups exhibited membrane blebbing, local shrinkage, and invagination, corresponding to early pyroptotic remodeling. The P1C1 group showed extensive membrane blebbing, irregular bubble formation, and even membrane rupture in some areas, indicating intense pyroptotic activity.

We further employed SEM to analyze surface structural changes (Figure 5C). SEM results were highly consistent with TEM observations. Control group cells had smooth, intact surfaces, while P1 and C1 treated cells exhibited membrane blebbing and bubble-like structures. The P1C1 group showed more severe surface damage, dense distribution of bubble-like protrusions, and blurred cell contours, consistent with late-stage pyroptotic morphology.

These observations confirmed from different perspectives that paclitaxel and cephalomannine combination induces severe membrane damage and accelerates cell death by synergistically activating pyroptosis-related mechanisms. We speculate that the two drugs not only activate apoptosis and necroptosis but also further trigger pyroptosis, forming a complex programmed cell death pattern. We subsequently conducted systematic analysis of key molecular markers involved in apoptosis, pyroptosis, and necroptosis pathways to fully elucidate their synergistic antitumor mechanisms.

### 3.5. Paclitaxel and Cephalomannine Induce Cell Death Signaling via ROS Accumulation-Mediated DNA Damage

Earlier research has demonstrated that drug-induced overproduction of ROS serves as a key upstream regulator in various cell death pathways [17]. ROS can disrupt mitochondrial function and induce apoptosis, as well as mediate DNA damage, thereby further activating pyroptosis, necroptosis, and other cell death pathways. Therefore, we investigated whether paclitaxel and cephalomannine induce oxidative stress, promote DNA damage, and activate downstream programmed cell death mechanisms.

**Figure 6:**
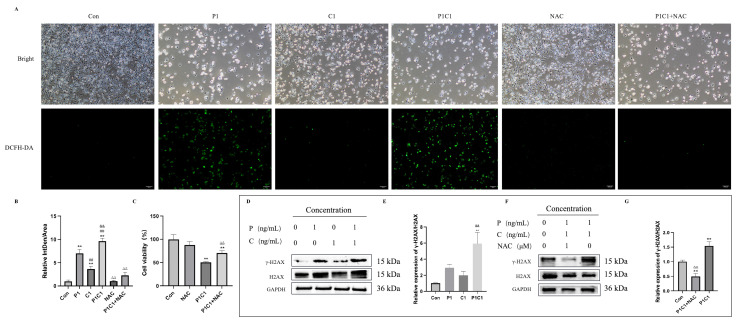
Paclitaxel and cephalomannine combination treatment induces DNA damage through ROS accumulation, which is partially reversed by NAC intervention (**A**–**G**). DCFH-DA fluorescence probe detection of intracellular ROS levels in MDA-MB-231 cells. Bright: bright field image; DCFH-DA: fluorescence image (**A**). Quantitative analysis of relative ROS fluorescence intensity (IntDen/Area) in different treatment groups (**B**). MTT assay showing changes in cell viability (**C**). Western blot analysis of γ-H2AX and H2AX protein expression (**D**). Quantification of relative γ-H2AX/H2AX expression ratio (**E**). Western blot analysis of γ-H2AX and H2AX expression after NAC intervention (**F**). Quantification of γ-H2AX/H2AX expression ratio after NAC intervention (**G**). Data are expressed as mean ± standard deviation (SD) from biological replicates (*n* = 3)., ** *p* < 0.01 vs. Con, ## *p* < 0.01 vs. P1, && *p* < 0.01 vs. C1, ∆∆ *p* < 0.01 vs. P1C1.

Intracellular ROS levels in MDA-MB-231 cells were detected using the DCFH-DA fluorescence probe to evaluate whether combination treatment enhanced oxidative stress. Fluorescence microscopy revealed that both P1 and C1 elevated ROS, while the P1C1 led to a markedly higher ROS level than either single treatment (Figure 6A). Quantitative analysis further confirmed that ROS fluorescence intensity was increased after P1 and C1 treatments, and was highest in the P1C1 group (Figure 6B).

Given that ROS may act as an upstream factor mediating DNA damage induced by the combination treatment, we used the ROS scavenger N-acetylcysteine (NAC) for validation. Fluorescence microscopy showed that NAC pretreatment significantly reduced ROS fluorescence intensity in the P1C1 group, bringing levels close to those of the control group (Figure 6A,B), confirming the central role of ROS in oxidative stress induced by the combination.

To explore whether ROS mediated DNA damage, we measured γ-H2AX expression levels. Western blot results showed that both P1 and C1 treatments upregulated γ-H2AX expression, with the highest levels observed in the P1C1 group (Figure 6D,E), indicating that combination treatment significantly enhanced DNA damage. Furthermore, NAC intervention effectively reduced γ-H2AX protein levels and its relative expression ratio (Figure 6F,G), thus confirming that paclitaxel and cephalomannine induce DNA damage via ROS accumulation.

Additionally, the combination treatment significantly decreased cell viability, which was partially restored by NAC intervention (Figure 6C), reinforcing the importance of DNA damage driven by oxidative stress in regulating cell death.

### 3.6. Paclitaxel and Cephalomannine Synergistically Promote Cell Apoptosis Through the p38/Caspase-3 Pathway and Mitochondria-Dependent Apoptotic Pathway

Apoptosis is a classical form of programmed cell death and involves the crosstalk of multiple signaling pathways. During this process, the p38 and p53 pathways promote the expression of pro-apoptotic protein Bax while suppressing the anti-apoptotic protein Bcl-2, leading to increased mitochondrial outer membrane permeability (MOMP) [18,19]. This alteration facilitates the release of Cytc from mitochondria [20], where it forms the apoptosome with Apaf-1 and other factors, triggering Caspase-9 activation and the initiation of the intrinsic apoptotic pathway [21]. Additionally, activation of external death receptors can trigger Caspase-8, thereby initiating the extrinsic apoptotic pathway. Additionally, activation of external death receptors can trigger Caspase-8, thereby initiating the extrinsic apoptotic pathway. Both Caspase-9 and Caspase-8 then activate Caspase-3 and Caspase-7 to execute apoptosis [22].

**Figure 7:**
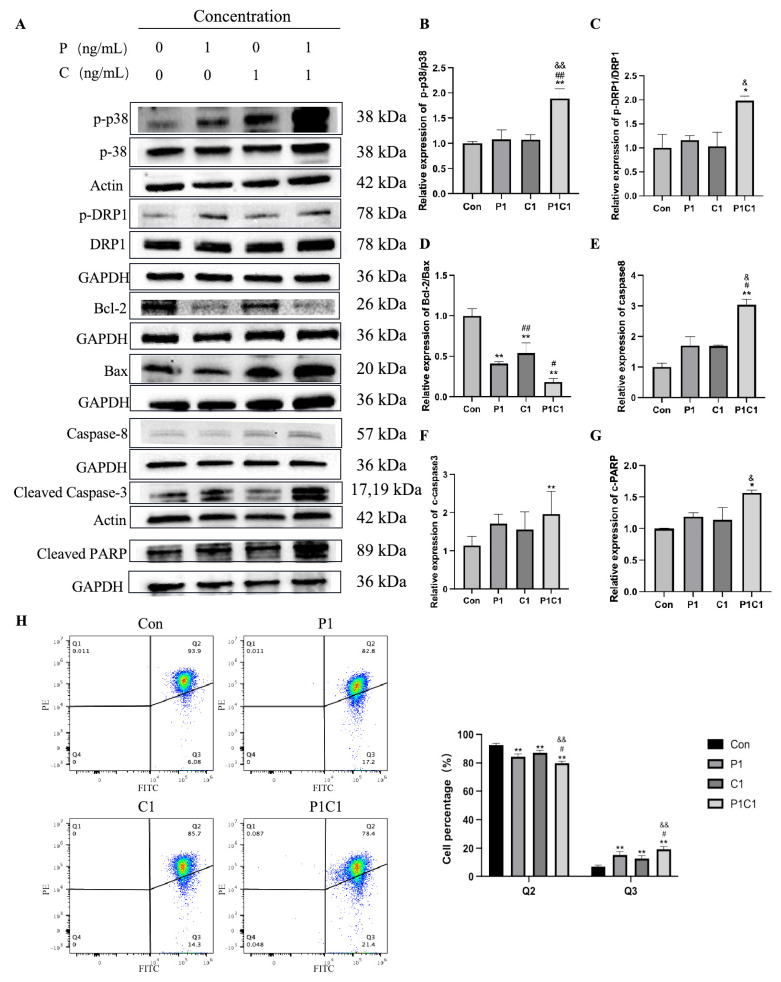
Effects of paclitaxel, cephalomannine, and their combination on the expression of apoptosis-related proteins (**A**–**H**). Western blot analysis of p-p38/p38, p-DRP1, Bcl-2, Bax, Caspase-8, Cleaved-Caspase-3, and Cleaved-PARP protein expression (**A**). Quantitative analysis of p-p38/p38 relative expression (**B**). Quantitative analysis of p-DRP1/DRP1 expression ratio (**C**). Quantitative analysis of Bcl-2/Bax expression ratio (**D**). Quantitative analysis of Caspase-8 expression (**E**). Quantitative analysis of Cleaved-Caspase-3 expression (**F**). Quantitative analysis of Cleaved-PARP expression (**G**). Measurement of mitochondrial membrane potential and result analysis (**H**). Data are expressed as mean ± standard deviation (SD) from biological replicates (*n* = 3). ** *p* < 0.01 vs. Con, * *p* < 0.05 vs. Con, ## *p* < 0.01 vs. P1, # *p* < 0.05 vs. P1, && *p* < 0.01 vs. C1, & *p* < 0.05 vs. C1.

Western blot analysis revealed that the combination treatment activated the p38 MAPK signaling pathway and upregulated p53 expression by jointly inducing oxidative stress and damage signals (Figure 4H and Figure 7B). Given that p38 requires phosphorylation for activation, we specifically examined p-p38 levels as an indicator of pathway activation. Further analysis combining Western blot and flow cytometry revealed that phosphorylation of mitochondrial dynamics protein DRP1 was increased in the combination group (Figure 7C), along with abnormal mitochondrial membrane potential (Figure 7H), both of which were much more pronounced than in single treatment groups. These results suggest that the combination treatment may induce mitochondrial dysfunction via activation of the p38 and p53 pathways, providing strong support for the occurrence of apoptosis. Subsequently, Bax expression was significantly upregulated and Bcl-2 expression significantly downregulated in the combination group compared with single treatments (Figure 7D). These findings suggest that paclitaxel and cephalomannine synergistically promote intrinsic apoptosis by modulating the Bax/Bcl-2 ratio and MOMP. Additionally, the combination treatment also activated extrinsic apoptotic signaling by increasing Caspase-8 enzymatic activity. Western blot analysis revealed a notable increase in Caspase-8 activation in the combination treatment group (Figure 7E), indicating that the combination effectively activated the extrinsic apoptotic pathway. Cleaved Caspase-3 levels were also markedly increased (Figure 7F). PARP, a key regulator of DNA damage repair and apoptosis, is cleaved by activated Caspase-3 during apoptosis, resulting in increased levels of cleaved PARP [23]. Notably, cleaved PARP levels were significantly elevated in the combination group (Figure 7G).

In conclusion, paclitaxel and cephalomannine combination treatment may lead to activation of phosphorylation and activation of p38, upregulation of p53 signaling, induction of mitochondrial dysfunction, promotion of Caspase activation, and cleavage of anti-apoptotic substrates such as PARP, ultimately resulting in the widespread apoptosis of tumor cells.

### 3.7. Paclitaxel and Cephalomannine Synergistically Promote Necroptosis via the RIPK1/RIPK3/MLKL Pathway

Necroptosis is a type of programmed cell death marked by cell membrane rupture and the release of intracellular contents, typically triggering local inflammation [24]. Activation of tumor necrosis factor receptor 1 (TNFR1) leads to the recruitment of TRADD and CYLD, which removes the ubiquitination of RIPK1 and promotes its interaction with RIPK3 to form the necrosome complex. The interaction between RIPK1 and RIPK3 facilitates the activation of the downstream molecule MLKL. During this process, DAMPs, such as HMGB1 and ATP, are released from the cell, amplifying local inflammatory responses and promoting necroptosis.

**Figure 8:**
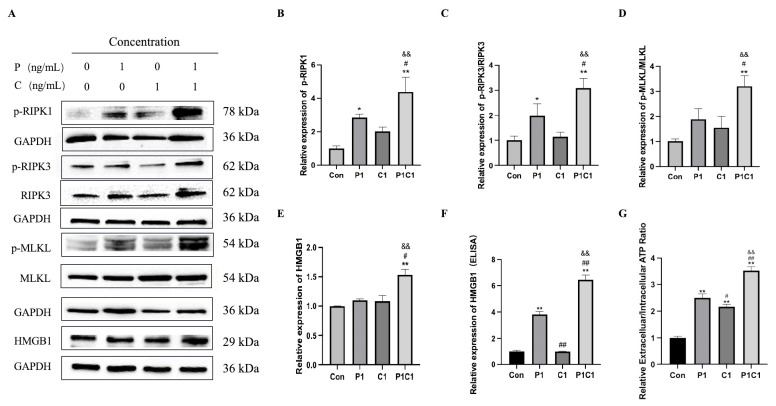
Effects of paclitaxel, cephalomannine, and their combination on the expression of necroptosis-related proteins (**A**–**G**). Western blot analysis of p-RIPK1, p-RIPK3, p-MLKL, and HMGB1 protein expression (**A**). Quantification of relative p-RIPK1 expression (**B**). Quantification of p-RIPK3/RIPK3 expression ratio (**C**). Quantification of p-MLKL/MLKL expression ratio (**D**). Quantification of intracellular HMGB1 expression (**E**). Quantification of HMGB1 levels in cell culture supernatant by ELISA (**F**). Relative extracellular/intracellular ATP ratio (**G**). Data are expressed as mean ± standard deviation (SD) from biological replicates (*n* = 3)., ** *p* < 0.01 vs. Con, * *p* < 0.05 vs. Con, ## *p* < 0.01 vs. P1, # *p* < 0.05 vs. P1, && *p* < 0.01 vs. C1.

We found that the phosphorylation levels of RIPK1 and RIPK3 (p-RIPK1 and p-RIPK3) were significantly increased in the P1C1 group compared with single drug treatments (Figure 8B,C), indicating synergistic activation of the RIPK1/RIPK3 pathway to drive necroptosis. RIPK3 subsequently phosphorylates and activates downstream MLKL, where it forms aggregates that compromise membrane integrity and cause the release of intracellular components [25]. Western blot analysis showed that phosphorylated MLKL levels were significantly increased in the combination group (Figure 8D), suggesting that the combination promoted MLKL activation and membrane localization, further driving membrane permeabilization and structural disintegration, ultimately resulting in necroptosis.

HMGB1 is a non-histone chromatin-binding protein primarily found in the nucleus. During necroptosis, it is released into the extracellular space, where it functions as a DAMP to trigger inflammatory signaling [26]. Protein expression analysis showed that combination treatment significantly increased intracellular HMGB1 expression (Figure 8E). ELISA analysis of the cell culture supernatant further confirmed that HMGB1 release into the extracellular environment was markedly increased after combination treatment (Figure 8F). These findings suggest that the combination not only induces necroptotic cell death but may also enhance immune responses in the tumor microenvironment, potentially improving therapeutic efficacy. We measured the ratio of extracellular to intracellular ATP as an indicator of membrane rupture and ATP leakage. The results showed that both paclitaxel and cephalomannine alone modestly increased extracellular ATP levels, while the combination treatment resulted in a significantly greater release of ATP (Figure 8G).

In summary, the combined treatment with paclitaxel and cephalomannine synergistically triggered oxidative stress and the release of damage-associated molecules such as HMGB1 and ATP, activated the RIPK1/RIPK3/MLKL signaling cascade, and promoted necroptosis in MDA-MB-231 cells.

### 3.8. Paclitaxel and Cephalomannine Regulate Cell Death via the NLRP3/Caspase-1/GSDMD Pyroptosis Pathway

Pyroptosis is a recently identified form of programmed cell death characterized by an inflammatory response [27]. Oxidative stress and damage signals induced by paclitaxel and cephalomannine can activate the assembly of the NLRP3 inflammasome, subsequently leading to the activation of Caspase-1 [28]. Activated Caspase-1 cleaves the precursor forms of pro-IL-1β and pro-IL-18 to generate mature IL-1β and IL-18, which mediate inflammatory responses. Simultaneously, the cleaved N-terminal fragment of GSDMD inserts into the plasma membrane to form pores, leading to membrane rupture, release of intracellular contents, and initiation of pyroptosis [29].

**Figure 9:**
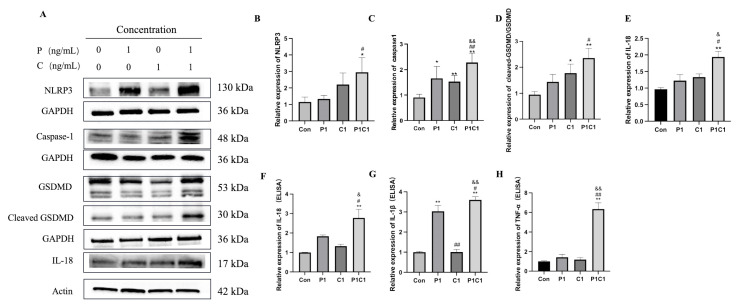
Mechanistic study of paclitaxel, cephalomannine, and their combination in regulating MDA-MB-231 cell death via activation of the pyroptosis pathway (**A**–**H**). Western blot analysis of NLRP3, Caspase-1, GSDMD and IL-18 protein expression (**A**). Quantification of relative NLRP3 expression (**B**). Quantification of relative Caspase1 expression (**C**). Quantification of cleaved-GSDMD/GSDMD expression ratio (**D**). Quantification of intracellular IL-18 expression (**E**). ELISA results of extracellular IL-18 levels (**F**). ELISA results of extracellular IL-1β levels (**G**). ELISA results of extracellular TNF-α levels (**H**). Data are expressed as mean ± standard deviation (SD) from biological replicates (*n* = 3). ** *p* < 0.01 vs. Con, * *p* < 0.05 vs. Con, ## *p* < 0.01 vs. P1, # *p* < 0.05 vs. P1, && *p* < 0.01 vs. C1, & *p* < 0.05 vs. C1.

As illustrated in Figure 9A,B, NLRP3 expression was markedly elevated following the combination treatment. Caspase-1 expression was also markedly higher in the combination group compared with single treatments (Figure 9C), promoting the formation of the pyroptosis-related complex and activation of downstream signals. Additionally, Figure 9D shows that the expression of cleaved GSDMD was significantly elevated following the combination treatment.

IL-18, a major inflammatory cytokine involved in pyroptosis, plays a crucial role in regulating cell death and antitumor immune responses [30]. As shown in Figure 9E,F, both Western blot and ELISA analyses demonstrated that the combination treatment significantly increased IL-18 expression and secretion levels. ELISA further showed that extracellular IL-1β and TNF-α secretion levels were significantly increased after combination treatment (Figure 9G,H). IL-1β and TNF-α are key pro-inflammatory cytokines that participate in pyroptosis and modulate immune responses [31]. Their levels were significantly higher in the combination group compared with single treatments, indicating that the combination not only promoted cell death through activation of the pyroptosis pathway but also enhanced the antitumor immune effect via activation of inflammatory responses.

In summary, paclitaxel and cephalomannine combination treatment may promote inflammatory cytokine release and pyroptosis through activation of the NLRP3/Caspase-1/GSDMD pathway.

### 3.9. Inhibitor-Based Validation of PANoptosis-Specific Regulatory Effects of Paclitaxel and Cephalomannine

Previous findings showed that paclitaxel and cephalomannine cooperatively triggered various forms of programmed cell death, including apoptosis, necroptosis, and pyroptosis, thereby inducing composite programmed cell death. To further confirm whether the observed effects of the combination were specifically mediated by PANoptosis, we introduced pathway-specific inhibitors for systematic evaluation of each pathway’s role in the combination treatment.

**Figure 10:**
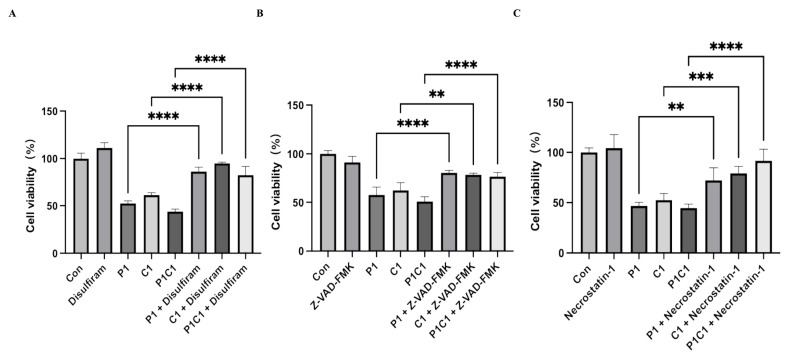
Effects of pyroptosis, apoptosis, and necroptosis inhibitors on cell viability following treatment with paclitaxel, cephalomannine, and their combination (**A**–**C**). Effect of the pyroptosis inhibitor Disulfiram on cell viability under different treatments (**A**). Effect of the apoptosis inhibitor Z-VAD-FMK on cell viability under different treatments (**B**). Effect of the necroptosis inhibitor Necrostatin-1 on cell viability under different treatments (**C**). P represents paclitaxel (P1 = 1 ng/mL); C represents cephalomannine (C1 = 1 ng/mL). Data are expressed as mean ± standard deviation (SD) from biological replicates (*n* = 5). **** *p* < 0.0001, *** *p* < 0.001, ** *p* < 0.01.

MDA-MB-231 cells were first pretreated with Z-VAD-FMK (apoptosis inhibitor), Necrostatin-1 (necroptosis inhibitor), or Disulfiram (pyroptosis inhibitor), and then exposed to the combined treatment of paclitaxel and cephalomannine (Figure 10A–C). The results showed that application of any of these inhibitors resulted in a partial reversal of drug-induced cell death in the P1C1 group, as evidenced by higher cell viability compared to the combination group without inhibitors. These findings confirm that apoptosis, pyroptosis, and necroptosis all play essential roles in mediating the synergistic cell death induced by paclitaxel and cephalomannine.

### 3.10. Validation of the Effects of Combination Treatment in Different TNBC Cell Lines and Normal Mammary Epithelial Cells

**Figure 11:**
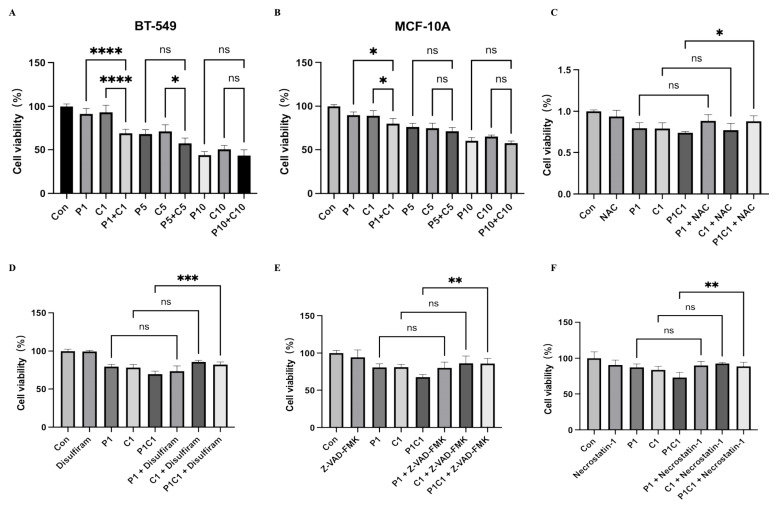
Validation of combination treatment effects and cell death mechanisms in BT-549 and MCF-10A cells. Effects of paclitaxel, cephalomannine, and their combination on cell viability in BT-549 and MCF-10A cells, and mechanistic investigation using pathway-specific inhibitors (**A**–**F**). Effect of paclitaxel, cephalomannine, and their combination at different concentrations on BT-549 cell viability (**A**). Effect of the same treatments on MCF-10A cell viability (**B**). Effect of the ROS scavenger NAC on cell viability under different treatments (**C**). Effect of the pyroptosis inhibitor Disulfiram on cell viability under different treatments (**D**). Effect of the apoptosis inhibitor Z-VAD-FMK on cell viability under different treatments (**E**). Effect of the necroptosis inhibitor Necrostatin-1 on cell viability under different treatments (**F**). P represents paclitaxel (P1 = 1 ng/mL); C represents cephalomannine (C1 = 1 ng/mL). Cell viability was measured using the MTT assay after 48 h of treatment. Data are expressed as mean ± standard deviation (SD) from biological replicates (*n* = 5). *** *p* < 0.0001, *** *p* < 0.001, ** *p* < 0.01, * *p* < 0.05.

To further evaluate the impact of combination treatment on the viability of different TNBC cell lines and assess its selectivity toward normal mammary epithelial cells, MTT assays were performed in BT-549 and MCF-10A cells. As shown in Figure 11A, paclitaxel combined with cephalomannine significantly inhibited cell viability in BT-549 cells at low concentrations (1 ng/mL), exhibiting a synergistic effect compared to monotherapy. Although BT-549 cells showed slightly lower sensitivity to single-agent treatments compared to the previously studied MDA-MB-231 cells, the combination treatment at the same dose still enhanced the cytotoxic effect effectively, indicating that this strategy may have general applicability across different TNBC subtypes.

MCF-10A, a non-tumorigenic normal mammary epithelial cell line, exhibited clear tolerance to low-dose treatment with P1, C1, or their combination P1C1 (Figure 11B), suggesting that the combined application of paclitaxel and cephalomannine exerts certain tumor selectivity. At higher concentrations, however, the treatment also caused a significant reduction in cell viability in MCF-10A cells, indicating that the combination regimen may exert a certain level of toxicity to normal cells under specific conditions, it is an observation consistent with previous reports [32].

Based on these findings, we further explored the potential mechanisms underlying the cytotoxicity induced by the combination treatment. Functional interference assays were performed in BT-549 cells using the ROS scavenger NAC, the apoptosis inhibitor Z-VAD-FMK, the pyroptosis inhibitor disulfiram, and the necroptosis inhibitor necrostatin-1 (Figure 11C–F). The results showed that NAC, disulfiram, Z-VAD-FMK, and necrostatin-1 all partially alleviated the reduction in cell viability induced by P+C treatment to varying degrees. These findings support the role of oxidative stress as a key upstream signal in triggering cell death and indicate the involvement of apoptosis, pyroptosis, and necroptosis, thereby further confirming that the combination treatment induces PANoptosis via activation of multiple coordinated pathways.

### 3.11. In Vivo Antitumor Effects of Paclitaxel and Cephalomannine Combination Therapy on Tumor Growth in a Xenograft Model

After confirming the synergistic inhibitory effects of paclitaxel and cephalomannine on tumor cell viability in vitro, we established a subcutaneous xenograft mouse model using MDA-MB-231 breast cancer cells to evaluate the antitumor effects of the combination therapy in vivo. The nude mice were randomly divided into four groups: control group (G1), paclitaxel group (G2), cephalomannine group (G3), and combination therapy group (G4, PC group).

**Figure 12:**
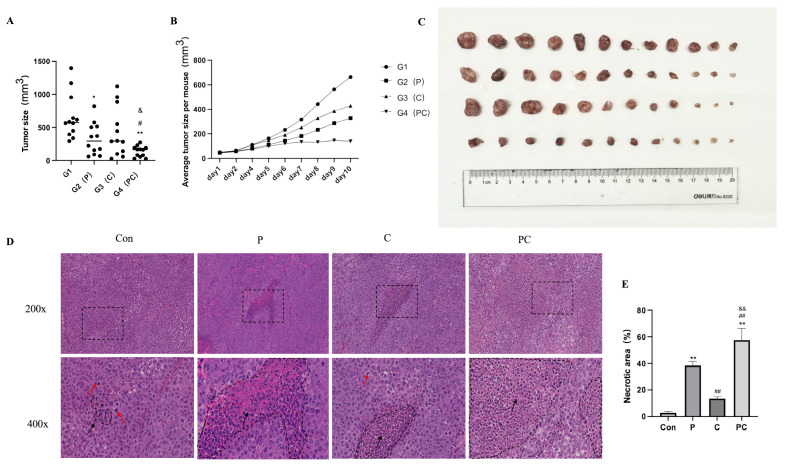
Comparison of tumor growth and tumor size in different treatment groups (**A**–**E**). Distribution of maximum tumor diameters (mm^3^) (**A**). Tumor volume progression over time as illustrated by growth curves (**B**). Photographs of excised tumors from each group (**C**). HE staining of tumor tissues from Con, P, C, and PC groups; dashed boxes indicate areas magnified in the lower panels (**D**). Quantification of tumor necrotic area (%) based on H&E staining (**E**). The data are presented in the form of mean ± standard deviation (SD). The analysis of tumor volume and size utilized the data from 12 mice in each group, while the quantification of necrotic areas was derived from histological analysis of 3 fields of view in each group, ** *p* < 0.01 vs. Con, * *p* < 0.05 vs. Con, ## *p* < 0.01 vs. P1, # *p* < 0.05 vs. P1, && *p* < 0.01 vs. C1, & *p* < 0.05 vs. C1.

Tumor size measurements showed that the combination therapy (G4) had a significant inhibitory effect on tumor growth. Both the maximum tumor diameter (Figure 12A) and the daily tumor volume trends (Figure 12B) indicated that combined administration of paclitaxel and cephalomannine significantly slowed tumor progression. Tumor volumes in the G4 group were markedly smaller than those in the control group, with the most pronounced suppression observed by day 10 of treatment compared to either monotherapy group. Photographic images of excised tumors further confirmed the efficacy of the combination treatment, with tumors in the G4 group appearing considerably smaller, demonstrating a superior antitumor effect. In contrast, single-agent treatments with paclitaxel or cephalomannine showed only limited tumor inhibition.

Histological examination by HE staining further supported these observations (Figure 12D,E). Tumors from the control group exhibited intact tissue structure, high cell density, tightly packed arrangements, high nuclear-to-cytoplasmic ratios, frequent pathological mitotic figures (red arrows), and minimal spontaneous necrosis (black arrows). The P group showed increased areas of necrosis, loose cytoplasm, and prominent vacuolization, with reduced pathological mitotic figures. The C group showed milder tissue disruption, relatively compact cell arrangements, and limited necrotic areas with active cell proliferation in some regions. The combination therapy group showed the most pronounced tumor damage: extensive necrosis and degeneration of tumor tissues, nuclear fragmentation or loss, disorganized residual tumor cells, loose cytoplasm, and no evident pathological mitotic figures, indicating that the cells were undergoing deep apoptosis or irreversible cell death.

**Figure 13:**
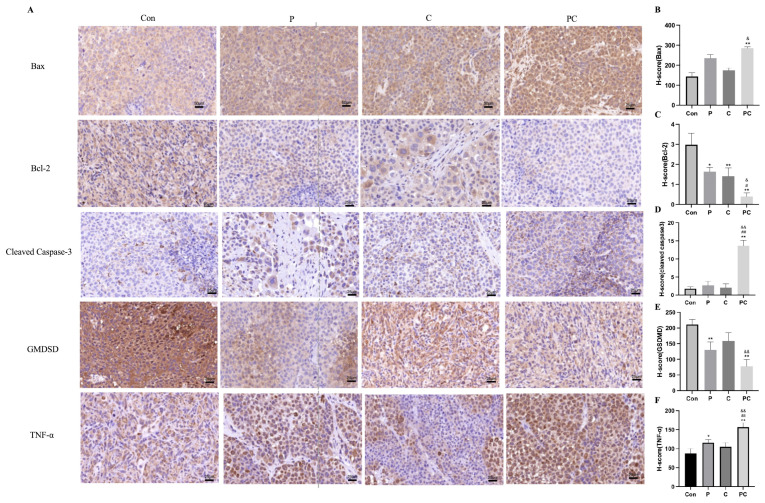
Immunohistochemical staining results of tumor tissues from the Con, P, C, and PC groups (**A**–**F**). Representative IHC images of Bax, Bcl-2, Cleaved Caspase-3, GSDMD, and TNF-α (**A**). Quantitative H-score analysis of Bax (**B**), Bcl-2 (**C**), Cleaved Caspase-3 (**D**), GSDMD (**E**), and TNF-α (**F**). Data are presented as mean ± standard deviation (*n* = 3), ** *p* < 0.01 vs. Con, * *p* < 0.05 vs. Con, ## *p* < 0.01 vs. P1, # *p* < 0.05 vs. P1, && *p* < 0.01 vs. C1, & *p* < 0.05 vs. C1.

Immunohistochemical staining was conducted to evaluate the expression of key proteins associated with cell death in tumor tissues (Figure 13A). The pro-apoptotic protein Bax showed low expression in the Con group, moderate positive expression in the single-agent groups, and the strongest, diffuse expression in the combination therapy group, indicating that the combination significantly enhanced apoptosis levels (Figure 13B). In contrast, The expression of Bcl-2 was the highest in the Con group, decreased in the single drug treatment group, and the lowest in the combined treatment group. This indicates that the combined treatment effectively inhibited the survival signal transduction of tumor cells (Figure 13C).

The apoptotic effector protein cleaved Caspase-3 exhibited strong positivity in the PC group, with intense brown-yellow staining in both the nucleus and cytoplasm, markedly higher than in the single-agent groups, indicating that the combination strongly activated the apoptotic cascade (Figure 13D). The pyroptosis-related protein GSDMD showed relatively high expression in the Con and C groups, likely representing the uncleaved precursor form; expression was slightly reduced in the P group and lowest in the PC group, suggesting that GSDMD had been cleaved and activated, initiating the pyroptosis process (Figure 13E).

The inflammatory cytokine TNF-α showed low expression in Con, gradually increased in the P and C group, and the strongest and most widespread positive expression in the PC group. This suggests that the combination treatment may have enhanced the TNFR1 signaling axis to activate the RIPK1/RIPK3 pathway, further driving necroptosis and promoting inflammatory responses within the tumor immune microenvironment (Figure 13F).

**Figure 14:**
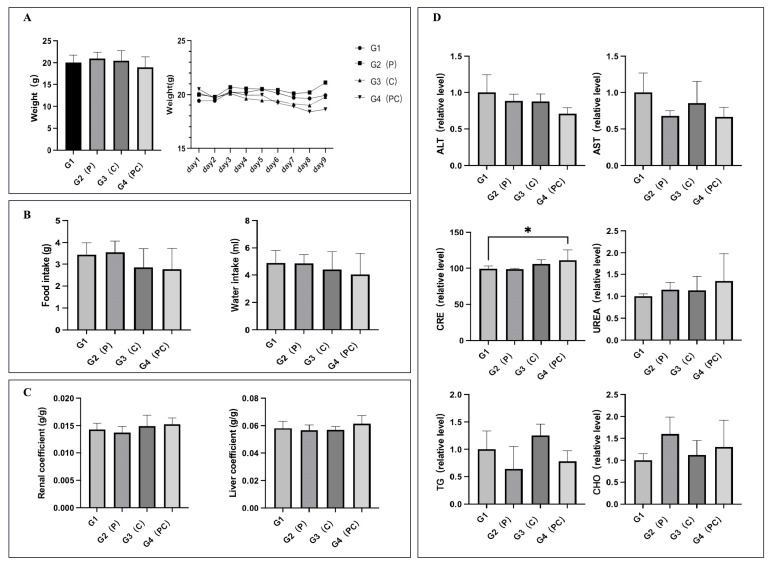
Safety evaluation of drug treatments in different groups (G1: control group, G2: paclitaxel group, G3: cephalomannine group, G4: combination therapy group, PC group) (**A**–**D**). Body weight change trends and statistical analysis among groups (**A**). Statistical analysis of food and water intake among groups (**B**). Statistical analysis of kidney and liver indices among groups (**C**). Statistical analysis of serum biochemical parameters among groups (**D**), including liver function (ALT, AST), kidney function (CRE, UREA), and lipid metabolism (TG, CHO). The data are presented in the form of mean ± standard deviation (SD) (*n* = 7), * *p* < 0.05 vs. Con.

Following the confirmation of the significant tumor inhibitory effect of paclitaxel and cephalomannine combination therapy, we also evaluated the systemic safety of this drug combination in vivo. The overall health status and key organ functions were comprehensively assessed by monitoring body weight, dietary behavior, liver and kidney indices, and serum biochemical parameters in nude mice (Figure 14).

All groups exhibited steady weight gain, and no weight loss was detected in the combination treatment group (Figure 14A). Moreover, there were no significant differences in food and water consumption across the groups (Figure 14B), indicating that the treatment did not affect basic feeding or metabolic behavior. Liver and kidney indices showed no significant variation among the groups (Figure 14C), suggesting that no pathological swelling or atrophy occurred and that the major metabolic organs remained functionally stable.

Serum biochemical results further supported these findings (Figure 14D). Levels of ALT and AST were slightly lower in the combination group compared to the control group, with no abnormal elevation, indicating that no hepatocellular injury occurred. Although CRE levels were slightly higher in the combination group and reached statistical significance, they remained within the normal physiological range; UREA levels showed no significant changes, indicating that renal function was not adversely affected. Lipid metabolism indicators, TG and CHO, showed no substantial differences between groups, demonstrating that the combination treatment did not cause dyslipidemia or metabolic abnormalities.

In conclusion, paclitaxel and cephalomannine combination therapy effectively inhibited tumor growth and activated multiple programmed cell death pathways without causing systemic toxicity or functional damage to key organs. These results demonstrate good in vivo tolerance and safety, providing a solid foundation for further clinical application.

## 4. Discussion

TNBC is recognized as the most challenging subtype of breast cancer to treat due to its high heterogeneity, strong invasiveness, and lack of specific targeted therapies. Clinically, paclitaxel is widely used to inhibit tumor cell division by disrupting microtubule dynamics; however, its efficacy is limited by the emergence of drug resistance and increased toxicity. Therefore, identifying combination strategies that can enhance the efficacy of paclitaxel and delay the development of resistance holds significant clinical value. Currently, several combination therapies are being explored in TNBC, such as paclitaxel combined with platinum-based drugs [33,34], immune checkpoint inhibitors [35,36], or antiangiogenic agents [37,38]. However, many of these regimens are limited by cumulative toxicity, lack of tumor selectivity, or modest clinical benefit. Compared with these approaches, our study introduces a novel combination strategy based on natural products that exhibits significant synergistic efficacy and multi-pathway activation, with promising biosafety.

In recent years, PANoptosis has emerged as a multifaceted form of regulated cell death that integrates multiple molecular targets and signaling pathways, as an important strategy for cancer cell treatment, yet effective drugs are still lacking. Previous studies have shown that paclitaxel not only induces tumor cell apoptosis but also activates necroptosis-related pathways to exert antitumor effects [39]. However, there have been no reports on paclitaxel-mediated pyroptosis. In this study, through integrated network pharmacology analysis and validation with in vitro and in vivo experiments, we systematically revealed for the first time from the perspective of PANoptosis the synergistic effects and potential molecular mechanisms of paclitaxel and cephalomannine combination therapy in TNBC, providing a novel therapeutic strategy and drug combination for clinical treatment. Notably, our study also revealed that the combination therapy induces intracellular accumulation of ROS and disrupts redox homeostasis, thereby activating multiple cell death pathways. This highlights the potential of natural products in modulating tumor oxidative stress and regulated cell death, aligning with the growing interest in ROS-mediated mechanisms and natural compound-based therapies within the field of antioxidant research.

Advances in systems biology have enabled network pharmacology and bioinformatics to become valuable tools for drug target prediction and mechanism exploration, leading to their widespread application [40]. In our study, network pharmacology analysis revealed that the common targets of paclitaxel and cephalomannine were mainly enriched in biological processes related to apoptosis, inflammatory responses, oxidative stress, and programmed cell death. The PPI network further identified core hub proteins including BCL2L1, MAPK14, SYK, TNF, and ADAM17, which play critical roles in the regulation of apoptosis, pyroptosis, and necroptosis, providing a clear direction for subsequent experimental validation. Molecular docking results confirmed these predictions. Both paclitaxel and cephalomannine were able to stably bind to these key targets, with complementary binding affinities. Paclitaxel exhibited stronger binding to necroptosis-associated targets such as TNF and ADAM17, while cephalomannine showed higher affinity toward apoptosis and inflammation-related targets including BCL2L1, MAPK14, and SYK. These findings suggest that the two compounds may act synergistically through distinct target pathways to construct a multi-pathway death network, resulting in a potential synergistic effect in the activation of programmed cell death signaling.

Subsequent experimental validation supported the above theoretical predictions. In this study, we found that the combination of paclitaxel and cephalomannine exerted significant synergistic anticancer activity in TNBC cells, including inhibition of cell proliferation, promotion of apoptosis, and suppression of cell migration. Cell viability assays demonstrated that even at low concentrations, the combination exhibited a strong synergistic inhibitory effect, with CI values less than 1, indicating synergism. Transmission and SEM further revealed multiple distinct morphological features of cell death following combination treatment, such as chromatin condensation associated with apoptosis, accumulation of autophagolysosomes, membrane blebbing, and rupture. These findings suggest that paclitaxel and cephalomannine may induce a composite programmed cell death process involving multiple death pathways [41].

Paclitaxel, cephalomannine, and other taxane derivatives promote tubulin polymerization, leading to DNA damage, inhibition of cell proliferation, and induction of apoptosis. Cell cycle analysis showed that combination treatment caused G2/M phase arrest and suppressed cell cycle progression via the p53/p21 axis, thereby further limiting the proliferative potential of tumor cells, which is consistent with previous findings. While both paclitaxel and cephalomannine are known to promote apoptosis, the specific types and molecular mechanisms of apoptosis remained unclear in earlier studies. Oxidative stress plays a key regulatory role in various forms of cell death [42]. In our study, paclitaxel and cephalomannine combination treatment significantly increased intracellular ROS levels and enhanced γ-H2AX expression. ROS accumulation not only induces DNA damage and triggers an unsuccessful DNA repair response but also acts as an upstream signal for multiple forms of programmed cell death [43]. ROS-mediated DNA damage further activated the p38/p53 axis and the mitochondrial apoptosis pathway, suggesting that the combination treatment initiated a comprehensive activation cascade from DNA damage to oxidative stress and ultimately to PANoptosis signaling. However, the detailed molecular mechanisms require further investigation.

Regarding apoptosis, oxidative stress and damage signals induced by paclitaxel and cephalomannine activated the p38 signaling pathway and upregulated p53 expression, leading to mitochondrial dysfunction (such as increased phosphorylation of the mitochondrial dynamics protein DRP1 and abnormal mitochondrial membrane potential) and promoting apoptosis. In this process, sequential activation of the p38 and p53 pathways upregulated the pro-apoptotic protein Bax while downregulating Bcl-2, thereby enhancing MOMP. This facilitated the release of Cytc from mitochondria into the cytoplasm, where free Cytc assembled with Apaf-1 and other components to form the apoptosome, subsequently activating Caspase-9. In parallel, the combination therapy also activated extrinsic death receptor pathways, leading to increased enzymatic activity of Caspase-8. The activated Caspase-9 and Caspase-8 then initiated the activation of downstream effector Caspases, including Caspase-3 and Caspase-7, which cleaved key substrates such as PARP and Lamin A/B to execute the apoptotic program [44].

Beyond classical apoptosis, necroptosis represents another key programmed cell death pathway with significant relevance to cancer treatment and tumor development [41]. The combination treatment activated necroptotic signaling pathways in cells, including TNFR1-mediated recruitment of TRADD and CYLD, which removed ubiquitin modifications from RIPK1 and facilitated the interaction between RIPK1 and RIPK3 to form the necrosome complex. Phosphorylated RIPK3 then activated downstream MLKL, which translocated to the plasma membrane and oligomerized to disrupt membrane integrity, leading to the release of intracellular contents. The released DAMPs, including HMGB1, ATP, and ROS, further amplified the local inflammatory response and induced programmed necrotic cell death.

Pyroptosis is also of great interest in the suppression of tumor cells, and regulation of pyroptosis signaling pathways and key molecular mediators is considered an important strategy for controlling tumor growth [45]. In this study, oxidative stress and damage signals induced by the combination treatment activated NLRP3 inflammasome assembly, which subsequently triggered the autocatalytic cleavage of pro-Caspase-1 into activated Caspase-1. Activated Caspase-1 cleaved precursor inflammatory cytokines pro-IL-1β and pro-IL-18 to generate mature IL-1β and IL-18, thereby mediating inflammatory responses. Additionally, Caspase-1 cleaves GSDMD to generate the N-terminal fragment, which relocates to the plasma membrane, forms pores, and causes cellular content release, swelling, and membrane rupture, thereby inducing pyroptosis. To verify the pathway specificity of this mechanism, we further introduced pathway-specific inhibitors for apoptosis (Z-VAD-FMK), pyroptosis (Disulfiram), and necroptosis (Necrostatin-1). The results showed that although all three inhibitors partially alleviated cell death, the combination treatment still exhibited a partial reversal effect under each condition. This outcome supports our hypothesis that the combination therapy induces cell death through multi-mechanism, multi-pathway PANoptosis regulation [7].

In BT-549 cells, the combination of paclitaxel and cephalomannine also exhibited a marked synergistic inhibitory effect, indicating that this therapeutic strategy possesses broad-spectrum efficacy and applicability across different TNBC subtypes. On the other hand, the normal mammary epithelial cell line MCF-10A demonstrated good tolerance to the combination treatment at low concentrations, suggesting a certain degree of tumor selectivity and potential safety.

In vivo experiments confirmed the antitumor efficacy of the combination therapy, as demonstrated by immunohistochemical analysis, which showed enhanced expression of pro-apoptotic proteins such as Bax and cleaved Caspase-3, along with decreased levels of Bcl-2 and precursor GSDMD. TNF-α expression was significantly upregulated, indicating that cell death signals may have been transduced into the tumor immune microenvironment to produce a synergistic antitumor response. Additionally, serum biochemical parameters, body weight, and liver and kidney indices showed no significant toxicity, indicating good safety and tolerability of the combination therapy.

Despite the comprehensive validation of the synergistic antitumor mechanisms of paclitaxel and cephalomannine from network prediction to in vitro and in vivo experiments, certain limitations remain. These include the lack of experimental validation of predicted network targets, the absence of exploration of additional forms of cell death, and limited investigation into other cancer types. Furthermore, the pharmacokinetics, such as bioavailability, and the impact of the combination therapy on the tumor immune microenvironment were not fully analyzed, nor were clinical samples evaluated. Although paclitaxel has been well studied and widely used in clinical practice, current research on cephalomannine is relatively limited. Most studies focus on its structural similarity to paclitaxel, while its pharmacological characteristics, toxicity profile, and translational potential remain poorly understood. Future studies are needed to further elucidate the molecular mechanisms and clinical translation of this combination strategy.

**Figure 15:**
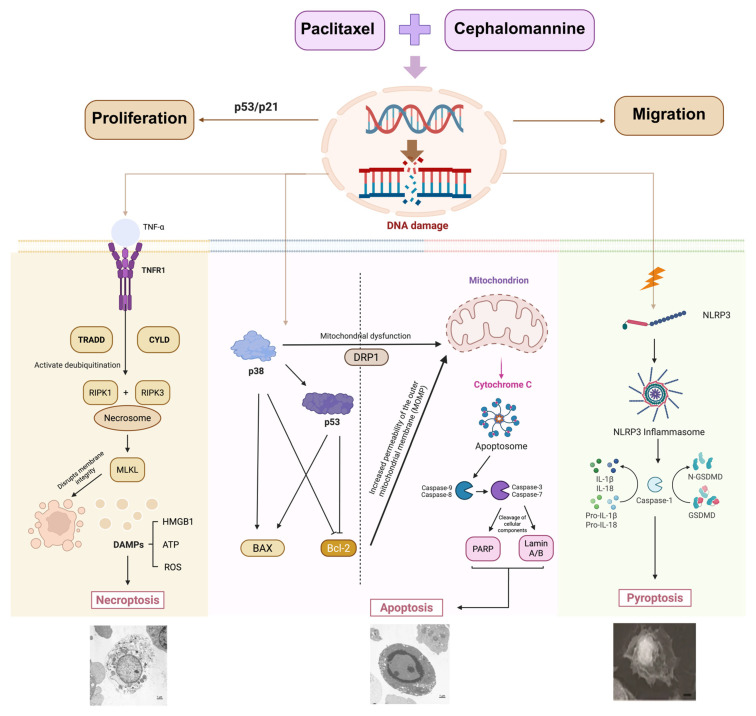
Proposed mechanism of the antitumor effects of paclitaxel and cephalomannine against TNBC.

In conclusion, this study is the first to systematically clarify the molecular mechanism by which paclitaxel and cephalomannine synergistically induce TNBC cell death through regulation of PANoptosis. A schematic of the proposed antitumor mechanism against TNBC is shown in Figure 15. The combination treatment initiated ROS-mediated oxidative stress, leading to DNA damage and activation of signaling cascades that synergistically activated multiple programmed cell death pathways, including mitochondrial apoptosis, necroptosis, and pyroptosis, to form a PANoptotic death network that suppressed proliferation and promoted tumor cell death. The combination also demonstrated strong antitumor efficacy and excellent biosafety in vivo. These findings provide important insights into the mechanisms of action of taxane-based anticancer drugs and offer valuable references for the development of novel PANoptosis-targeted anticancer therapies.

## 5. Conclusions

In conclusion, this study comprehensively demonstrated that the combination of paclitaxel and cephalomannine exerts potent synergistic antitumor effects in triple-negative breast cancer through the coordinated activation of apoptosis, necroptosis, and pyroptosis pathways. This PANoptosis-inducing mechanism is driven by ROS accumulation, DNA damage, and mitochondrial dysfunction, as supported by both cellular and molecular evidence. Importantly, the combination treatment also showed strong efficacy and good safety profiles in vivo, as reflected by tumor suppression and the absence of significant toxicity in major organs. These findings provide a solid foundation for further investigation into the pharmacological properties and translational potential of cephalomannine-based therapies in the context of TNBC.

## Figures and Tables

**Table 1:** Binding energy data of paclitaxel and cephalomannine with BCL2L1, MAPK14, SYK, TNF, and ADAM17 proteins in molecular docking analysis.

Drug	Target	Binding Energy (-kcal/mol)	PDB ID
Paclitaxel	BCL2L1	7.4	7JGW
Paclitaxel	MAPK14	7.9	3OEF
Paclitaxel	SYK	8.6	4YJR
Paclitaxel	TNF	9.3	1EXT
Paclitaxel	ADAM17	9.5	2FV5
Cephalomannine	BCL2L1	7.9	7JGW
Cephalomannine	MAPK14	9.0	3OEF
Cephalomannine	SYK	8.7	4YJR
Cephalomannine	TNF	7.4	1EXT
Cephalomannine	ADAM17	8.2	2FV5

**Table 2:** Combination index (CI) of paclitaxel and cephalomannine (1:1 ratio) at different total concentrations.

Total Concentration of Paclitaxel + Cephalomannine (ng/mL)	Fa (Fraction Affected) (%)	CI
2	44	0.13533
2	40	0.09889
2	39	0.09137
10	38	0.42198
10	37	0.38959
10	42	0.57862
20	34	0.61104
20	36	0.71899
20	35	0.66305

## Data Availability

The original contributions presented in this study are included in the article. Further inquiries can be directed to the corresponding author.

## References

[1] Katsura C., Ogunmwonyi I., Kankam H.K., Saha S. (2022). Breast cancer: Presentation, investigation and management. Br. J. Hosp. Med..

[2] Singh D.D., Yadav D.K. (2021). TNBC: Potential Targeting of Multiple Receptors for a Therapeutic Breakthrough, Nanomedicine, and Immunotherapy. Biomedicines.

[3] Long L., Fei X., Chen L., Yao L., Lei X. (2024). Potential therapeutic targets of the JAK2/STAT3 signaling pathway in triple-negative breast cancer. Front. Oncol..

[4] Obidiro O., Battogtokh G., Akala E.O. (2023). Triple Negative Breast Cancer Treatment Options and Limitations: Future Outlook. Pharmaceutics.

[5] Dai X., Deng Z., Liang Y., Chen L., Jiang W., Zhao W. (2020). Enterococcus faecalis induces necroptosis in human osteoblastic MG63 cells through the RIPK3/MLKL signalling pathway. Int. Endod. J..

[6] Tsunemitsu Y., Kagawa S., Tokunaga N., Otani S., Umeoka T., Roth J.A., Fang B., Tanaka N., Fujiwara T. (2004). Molecular therapy for peritoneal dissemination of xenotransplanted human MKN-45 gastric cancer cells with adenovirus mediated Bax gene transfer. Gut.

[7] Sun X., Yang Y., Meng X., Li J., Liu X., Liu H. (2024). PANoptosis: Mechanisms, biology, and role in disease. Immunol. Rev..

[8] Zhang X., Tang B., Luo J., Yang Y., Weng Q., Fang S., Zhao Z., Tu J., Chen M., Ji J. (2024). Cuproptosis, ferroptosis and PANoptosis in tumor immune microenvironment remodeling and immunotherapy: Culprits or new hope. Mol. Cancer.

[9] Gao X., Guo Y., Chen K., Wang H., Xie W. (2024). Study on the Chemical Constituents, Pharmacological Activities, and Clinical Application of Taxus. Am. J. Chin. Med..

[10] Gao X., Zhang N., Xie W. (2024). Advancements in the Cultivation, Active Components, and Pharmacological Activities of Taxus mairei. Molecules.

[11] Chabner B. (1991). Taxol. Principles & Practice of Oncology Update.

[12] Sekar P., Ravitchandirane R., Khanam S., Muniraj N., Cassinadane A.V. (2022). Novel molecules as the emerging trends in cancer treatment: An update. Med. Oncol..

[13] An S., Xu X., Bao Y., Su F., Jiang Y. (2024). Cephalomannine reduces radiotherapy resistance in non-small cell lung cancer cells by blocking the β-catenin-BMP2 signaling pathway. Tissue Cell.

[14] Wang X., Li J., Chen R., Li T., Chen M. (2023). Active Ingredients from Chinese Medicine for Combination Cancer Therapy. Int. J. Biol. Sci..

[15] Pinzi L., Rastelli G. (2019). Molecular Docking: Shifting Paradigms in Drug Discovery. Int. J. Mol. Sci..

[16] Levine A.J. (1997). p53, the cellular gatekeeper for growth and division. Cell.

[17] Fernandes G.M.M., Serafim Junior V., Galbiatti-Dias A.L.S., Ferreira L.A.M., Castanhole-Nunes M.M.U., Kawasaki-Oyama R.S., Maniglia J.V., Pavarino E.C., Goloni-Bertollo E.M. (2022). Treatment effects of the EGFR pathway drugs on head and neck cancer stem cells. Am. J. Cancer Res..

[18] Huang Y.K., Chang K.C., Li C.Y., Lieu A.S., Lin C.L. (2023). AKR1B1 Represses Glioma Cell Proliferation through p38 MAPK-Mediated Bcl-2/BAX/Caspase-3 Apoptotic Signaling Pathways. Curr. Issues Mol. Biol..

[19] Chen Y.T., Lin C.W., Su C.W., Yang W.E., Chuang C.Y., Su S.C., Hsieh M.J., Yang S.F. (2021). Magnolol Triggers Caspase-Mediated Apoptotic Cell Death in Human Oral Cancer Cells through JNK1/2 and p38 Pathways. Biomedicines.

[20] Hassin O., Oren M. (2023). Drugging p53 in cancer: One protein, many targets. Nat. Rev. Drug Discov..

[21] Yuan S., Akey C.W. (2013). Apoptosome structure, assembly, and procaspase activation. Structure.

[22] Gryko M., Łukaszewicz-Zając M., Guzińska-Ustymowicz K., Kucharewicz M., Mroczko B., Algirdas U. (2023). The caspase-8 and procaspase-3 expression in gastric cancer and non-cancer mucosa in relation to clinico-morphological factors and some apoptosis-associated proteins. Adv. Med. Sci..

[23] Kadam A., Jubin T., Roychowdhury R., Begum R. (2020). Role of PARP-1 in mitochondrial homeostasis. Biochim. Biophys. Acta (BBA)-Gen. Subj..

[24] Rickard J.A., O’Donnell J.A., Evans J.M., Lalaoui N., Poh A.R., Rogers T., Vince J.E., Lawlor K.E., Ninnis R.L., Anderton H. (2014). RIPK1 regulates RIPK3-MLKL-driven systemic inflammation and emergency hematopoiesis. Cell.

[25] Ai Y., Meng Y., Yan B., Zhou Q., Wang X. (2024). The biochemical pathways of apoptotic, necroptotic, pyroptotic, and ferroptotic cell death. Mol. Cell.

[26] Huang W., Tang Y., Li L. (2010). HMGB1, a potent proinflammatory cytokine in sepsis. Cytokine.

[27] Li S., Sun Y., Song M., Song Y., Fang Y., Zhang Q., Li X., Song N., Ding J., Lu M. (2021). NLRP3/caspase-1/GSDMD-mediated pyroptosis exerts a crucial role in astrocyte pathological injury in mouse model of depression. JCI Insight.

[28] Yu T., Di G. (2017). Role of tumor microenvironment in triple-negative breast cancer and its prognostic significance. Chin. J. Cancer Res..

[29] Wang C., Yang T., Xiao J., Xu C., Alippe Y., Sun K., Kanneganti T.D., Monahan J.B., Abu-Amer Y., Lieberman J. (2021). NLRP3 inflammasome activation triggers gasdermin D-independent inflammation. Sci. Immunol..

[30] Becker-Hapak M.K., Shrestha N., McClain E., Dee M.J., Chaturvedi P., Leclerc G.M., Marsala L.I., Foster M., Schappe T., Tran J. (2021). A Fusion Protein Complex that Combines IL-12, IL-15, and IL-18 Signaling to Induce Memory-Like NK Cells for Cancer Immunotherapy. Cancer Immunol. Res..

[31] Kapoor M., Martel-Pelletier J., Lajeunesse D., Pelletier J.P., Fahmi H. (2011). Role of proinflammatory cytokines in the pathophysiology of osteoarthritis. Nat. Rev. Rheumatol..

[32] Lim S.J., Choi H.G., Jeon C.K., Kim S.H. (2015). Increased chemoresistance to paclitaxel in the MCF10AT series of human breast epithelial cancer cells. Oncol. Rep..

[33] Yu K.D., Ye F.G., He M., Fan L., Ma D., Mo M., Wu J., Liu G.Y., Di G.H., Zeng X.H. (2020). Effect of Adjuvant Paclitaxel and Carboplatin on Survival in Women With Triple-Negative Breast Cancer: A Phase 3 Randomized Clinical Trial. JAMA Oncol..

[34] Saloustros E., Nikolaou M., Kalbakis K., Polyzos A., Christofillakis C., Kentepozidis N., Pistamaltzian N., Kourousis C., Vamvakas L., Georgoulias V. (2018). Weekly Paclitaxel and Carboplatin Plus Bevacizumab as First-Line Treatment of Metastatic Triple-Negative Breast Cancer. A Multicenter Phase II Trial by the Hellenic Oncology Research Group. Clin. Breast Cancer.

[35] Corti C., Koca B., Rahman T., Mittendorf E.A., Tolaney S.M. (2025). Recent Advances in Immune Checkpoint Inhibitors for Triple-Negative Breast Cancer. ImmunoTargets Ther..

[36] Yardley D.A., Coleman R., Conte P., Cortes J., Brufsky A., Shtivelband M., Young R., Bengala C., Ali H., Eakel J. (2018). nab-Paclitaxel plus carboplatin or gemcitabine versus gemcitabine plus carboplatin as first-line treatment of patients with triple-negative metastatic breast cancer: Results from the tnAcity trial. Ann. Oncol..

[37] Chen M., Huang R., Rong Q., Yang W., Shen X., Sun Q., Shu D., Jiang K., Xue C., Peng J. (2025). Bevacizumab, tislelizumab and nab-paclitaxel for previously untreated metastatic triple-negative breast cancer: A phase II trial. J. Immunother. Cancer.

[38] Miller K., Wang M., Gralow J., Dickler M., Cobleigh M., Perez E.A., Shenkier T., Cella D., Davidson N.E. (2007). Paclitaxel plus bevacizumab versus paclitaxel alone for metastatic breast cancer. N. Engl. J. Med..

[39] Gielecińska A., Kciuk M., Yahya E.B., Ainane T., Mujwar S., Kontek R. (2023). Apoptosis, necroptosis, and pyroptosis as alternative cell death pathways induced by chemotherapeutic agents?. Biochim. Biophys. Acta (BBA)-Rev. Cancer.

[40] Nogales C., Mamdouh Z.M., List M., Kiel C., Casas A.I., Schmidt H. (2022). Network pharmacology: Curing causal mechanisms instead of treating symptoms. Trends Pharmacol. Sci..

[41] D’Arcy M.S. (2019). Cell death: A review of the major forms of apoptosis, necrosis and autophagy. Cell Biol. Int..

[42] Donohoe C., Senge M.O., Arnaut L.G., Gomes-da-Silva L.C. (2019). Cell death in photodynamic therapy: From oxidative stress to anti-tumor immunity. Biochim. Biophys. Acta (BBA)-Rev. Cancer.

[43] Nakamura T., Naguro I., Ichijo H. (2019). Iron homeostasis and iron-regulated ROS in cell death, senescence and human diseases. Biochim. Biophys. Acta (BBA)-Gen. Subj..

[44] Elmore S. (2007). Apoptosis: A review of programmed cell death. Toxicol. Pathol..

[45] Fang Y., Tian S., Pan Y., Li W., Wang Q., Tang Y., Yu T., Wu X., Shi Y., Ma P. (2020). Pyroptosis: A new frontier in cancer. Biomed. Pharmacother..

